# Novel Biomimetic “Spider Web” Robust, Super‐Contractile Liquid Crystal Elastomer Active Yarn Soft Actuator

**DOI:** 10.1002/advs.202400557

**Published:** 2024-02-28

**Authors:** Dingsheng Wu, Xin Li, Yuxin Zhang, Xinyue Cheng, Zhiwen Long, Lingyun Ren, Xin Xia, Qingqing Wang, Jie Li, Pengfei Lv, Quan Feng, Qufu Wei

**Affiliations:** ^1^ Key Laboratory of Eco‐Textiles, Ministry of Education Jiangnan University Jiangsu 214122 China; ^2^ Key Laboratory of Textile Fabrics, College of Textiles and Clothing Anhui Polytechnic University Anhui 241000 China; ^3^ College of Textile and Clothing Xinjiang University Urumchi Xinjiang 830046 China; ^4^ Jiangsu Textile Quality Services Inspection Testing Institute Jiangsu 210007 China

**Keywords:** active yarn actuator, electrospinning, liquid crystal elastomer, smart fabrics, super‐contractile

## Abstract

In nature, spider web is an interwoven network with high stability and elasticity from silk threads secreted by spider. Inspired by the structure of spider webs, light‐driven liquid crystal elastomer (LCE) active yarn is designed with super‐contractile and robust weavability. Herein, a novel biomimetic gold nanorods (AuNRs) @LCE yarn soft actuator with hierarchical structure is fabricated by a facile electrospinning and subsequent photocrosslinking strategies. Meanwhile, the inherent mechanism and actuation performances of the as‐prepared yarn actuator with interleaving network are systematically analyzed. Results demonstrate that thanks to the unique “like‐spider webs” structure between fibers, high molecular orientation within the LCE microfibers and good flexibility, they can generate super actuation strain (≈81%) and stable actuation performances. Importantly, benefit from the robust covalent bonding at the organic–inorganic interface, photopolymerizable AuNRs molecules are uniformly introduced into the polymer backbone of electrospun LCE yarn to achieve tailorable shape‐morphing under different light intensity stimulation. As a proof‐of‐concept illustration, light‐driven artificial muscles, micro swimmers, and hemostatic bandages are successfully constructed. The research disclosed herein can offer new insights into continuous production and development of LCE‐derived yarn actuator that are of paramount significance for many applications from smart fabrics to flexible wearable devices.

## Graphical abstract

A super‐contractile gold nanorods@liquid crystal elastomeractive yarn with “like‐spider webs” hierarchical structure is continuously fabricated by a simple electrospinning strategy. Thanks to the flexible and weavability properties of active yarn, the light‐controlled artificial muscle, micro‐swimmer, and smart hemostatic bandage is designed and constructed.

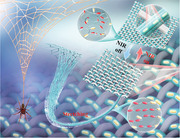

## 1 Introduction

Fibers soft actuator can be found in nature, and thanks to their excellent anisotropic contraction and perceptua feature, enable the effective information transmission and self‐protection for living creatures.^[^ ^1^, ^2^, ^3^ ^]^ For instance, spider silk, venus flytrap, and mammalian muscles can rapidly contract and deform under external environmental stimulation, which is used to capture food and exchange information.^[^ ^4^, ^5^, ^6^, ^7^ ^]^ Inspired by the above biological actuation systems, many scientists have made great efforts to fabricate soft actuators with contractile functionalities in response to external stimuli, exhibiting potential applications in the field of intelligent wearable, soft robot, energy information transmission.^[^ ^8^, ^9^ ^]^ As a typical intelligent stimulus‐response material within soft actuator, liquid crystal elastomer (LCE) is a kind of light cross‐linked polymer with unique bidirectional shape memory function, which combines the self‐organization and responsiveness of liquid crystal units with the entropy elasticity of polymer rubber.^[^ ^10^, ^11^, ^12^, ^13^ ^]^ Among them, LCE fiber, a common form of LCE, has a large shape variable and good weavability under external stimulation, endowing them diverse applications in artificial muscles, smart textiles and soft robots.^[^ ^14^, ^15^, ^16^ ^]^ It is well known that LCE fibers are mainly prepared by using liquid crystal monomers or liquid crystal polymer precursors mixed with cross‐linking agents to form cross‐linked network structured polymers, which are formed into fibers of different diameters by direct ink writing, wet spinning, electrostatic spinning and three‐dimensional printing strategies.^[^ ^17^, ^18^ ^]^ Among them, the high voltage electrospinning technique as one of the most common and convenient methods for the preparation of continuous micro/nano fibers.^[^ ^19^, ^20^ ^]^ Recent researches have demonstrated that the electrostatic spinning technology can be used to continuously produce uniform LCE micro/nano fibers with small fiber diameter, fast response time and high energy density, which have good prospects for application in the field of micro‐oscillators, soft actuator.^[^ ^21^, ^22^ ^]^

Among the many external stimuli, light and heat are two typical clean energy sources that have the advantages of being widely available, low cost and highly controllable.^[^ ^23^, ^24^, ^25^, ^26^ ^]^ Compared with the thermal stimulation actuating of the LCE, the photo‐stimulation response process usually requires that the LCE contains mediator that can absorb light energy and efficiently convert into heat energy.^[^ ^27^ ^]^ The internal structure of the molecule undergoes a reversible photoisomerization or photothermal reaction under light irradiation, which induces a phase transition behavior of the LCE from the liquid crystal phase to each homogeneous phase, achieving them shape‐morphing.^[^ ^28^ ^]^ Lv et al. found that the extrusion spinning strategy was used to quickly prepare a variety of light‐driven fiber actuators, showing excellent actuation strain, actuation time and mechanical properties, which provided a good strategy for the practical application of LCE in soft actuator.^[^ ^29^ ^]^ Terentjev et al. developed thermoplastic liquid crystal elastomer fine fibers via using melt spinning technology, which possessed a high actuation deformation rate, but their weaving performance and reusability need to be further enhanced.^[^ ^30^ ^]^ Cai et al. prepared a LCE microfiber actuators via using electrospun technology. Compared with other actuators, microfiber actuators exhibited large actuation strain with a fast response speed.^[^ ^21^ ^]^ Yang et al. designed a twisted LCE fiber to achieve untethered and reversible responsiveness and noted that the torsional fiber actuator successfully cuts the magnetic induction line and generates a stable current under thermal stimulation.^[^ ^31^ ^]^ Recently, our group has constructed a novel photothermal multi‐stimulus response flexible smart optoelectronic devices using the MXene‐modified LCE fiber actuator.^[^ ^32^ ^]^ The photo‐responsive liquid crystal elastomer fibers can be remotely manipulated by changing the position, wavelength and intensity of the light under light stimulation. Numerous studies revealed that adding various inorganic nanomaterials (e.g., graphene, carbon nanotubes, liquid metals, and gold nanorods) to LCE fibers can achieve their responsive actuating ability to light and thermal stimuli. Among them, gold nanorods (AuNRs) have been considered as one of the most promising photothermal nanoagents due to their superior capability of efficiently converting light energy into localized heat through a non‐radiative relaxation process known as localized surface plasmon resonances.^[^ ^33^ ^]^ However, there are still some defects in light‐driven inorganic nanomaterials doped LCE fiber actuator that limit its further application, such as the complex production processes, the difficulty of limited deformation rate and the insufficient binding ability between inorganic photothermal agents and organic interfaces.

Interestingly, the natural spider webs are elastic and fibrous interweaving webs made by spiders to catch food, protect itself, and transmit information.^[^ ^34^ ^]^ When the external force acts on the spider web, not only the single fiber in the web will deform correspondingly, but also the shape of the fiber network will deform, thereby dispersing the buffer force and achieving a large deformation effect.^[^ ^29^ ^]^ This structure is like a spring network, which not only has the elasticity of a single spring, but also shows elastic characteristics of the fiber network changes. Moreover, this structure can also maintain a certain tension and stability of the spider web, and will not relax or collapse due to wind or gravity. Inspired by natural spider web with large deformation and stable structure, we fabricate light‐driven gold nanorods‐doped LCE (AuNRs@LCE) soft actuator with cross‐scale hierarchical structure (from liquid crystal molecules, fiber interwoven network to active orientation yarn) and super‐contractile via a combination of electrospinning and subsequent photocrosslinking strategies. This method not only endows the fibers actuators with good photothermal stimulation response and super‐contractile performance, but also ensures that the stable structure and excellent mechanical flexibility of the fiber actuator. Importantly, the introduction of photopolymerisable AuNRs into the molecular chain of LCE polymers by the liquid crystal elastomer synthesis process not only provides a new idea for the introduction of inorganic nanoparticles into the organic polymer system, but also improves the interfacial bond strength between organic‐inorganic materials through the generation of robust chemical covalent bonds. Moreover, thanks to the high orientation of mesogenic unit within the fiber and the advantage of inter‐fiber hierarchical structure (from liquid crystal molecules, fiber interwoven network to active orientation yarn), the as‐prepared AuNRs@LCE active yarn exhibit excellent actuacting performances to light or thermal stimulation, and its actuacting rate is much higher than most reported literature. Furthermore, the active yarn has good weaveable properties and excellent mechanical strength during preparation, making them diverse applications prospects. As a proof‐of‐concept demonstration, the designed AuNRs@LCE active soft actuator proved to be capable of performing various precise and remote photo‐thermal response locomotions, such as a light controlled super contraction artificial muscle, micro swimmers, and hemostatic bandages. The hierarchical structure design strategy adopted by the research opens up a new avenue that could lead to the development of stable, large deformation and super‐contractile LCE fiber soft actuator for emerging applications ranging from artificial muscles to soft robots and smart wearables.

## 2 Results and Discussion

**Figure 1:**
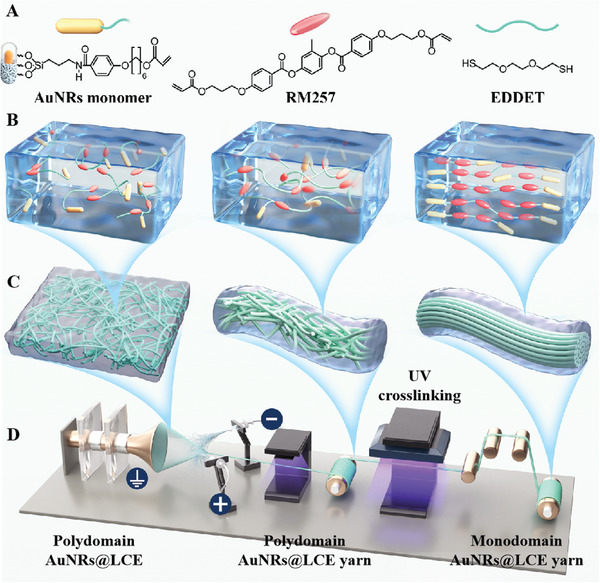
A) Molecular structural of monomers and chain extenders; B,C) Schematic diagram of internal molecular chain and fibers network structure changes in the electrospun yarn soft actuator; D) Schematic diagram of the preparation process of the AuNRs@LCE active yarn soft actuator.

Herein, inspired by natural spider web with large deformation and stable structure, a novel smart yarn soft actuator that consisting of scalable AuNRs@LCE fibers was manufactured by using a combination of high voltage electrospinning technology, molecular synthesis and a two‐step cross‐linking strategy, as shown in **Figure** **1**. Prior to electrospinning, we synthesized numerous AuNRs with excellent photo‐thermal conversion efficiency and uniform size as photothermal additives in this work. In order to assure the uniform dispersion and stable interface bonding effect of inorganic photothermal conversion molecule (AuNRs) in organic intelligent driving materials (LCE), we wisely designed and synthesized an unique polymerizable AuNRs nanomonomer via using uniform salinization modification method. The preparation diagrammatic sketch of polymerizable AuNRs nanomonomer was shown in Figure S1A (Supporting Information). And the detailed synthesis and functionalization modification process of AuNRs monomer can be found in the Supporting Information. Subsequently, we conveniently prepared a homogeneous and stable liquid crystal oligomer spinning solution, in which 1,4‐bis‐[4‐(6‐acryloyloxyhexyloxy) benzoyloxy]−2‐methylbenzene (RM257), 2,2′‐dipropylamine (DPA), (ethylenedioxy)diethanethiol (EDDET), and 2‐hydroxy‐4′‐(2‐hydroxyethoxy)−2‐methylpropiophenone (HHMP) were used as liquid crystal monomers, catalysts, chain extenders, and photoinitiators in the reaction system, respectively. During the preparation of the spinning solution, the carbon‐carbon double bonds in the liquid crystal monomer (RM257) and AuNRs monomer can undergo the first step crosslinking reaction (Michael addition reaction) with the mercapto group in the chain extender (EDDET) under the catalyst, forming a short chain liquid crystal oligomer. In the subsequent UV crosslinking, the carbon‐carbon double bonds at the end group of the molecular chain or with the remaining AuNRs monomer undergo a double bond addition reaction, The distribution state of its molecular chains and the binding mode of its liquid crystal components are shown in Figure 1A,B.

Afterward, as shown in Figure 1C, an electrospinning solution consisting of liquid crystal oligomer dissolved in dichloromethane (CH_2_Cl_2_) solvent was directly electrospun from two individual nozzles and the electrospun nanofibers yarn was wound onto a PTFE roller thread by rotating the funnel collector. The extrusion speed of both propulsion pumps and receiving distance of the spinning solution were set to 1.2 ml h^−1^ and 6–8 cm during the electrospinning process. Meanwhile, two spinnerets were maintained at positive (10 kV) and negative potentials (−8 kV), respectively, and the metal funnel‐shaped receiver was kept grounded. Due to the stable voltage distribution between the two spinnerets and the metal funnel‐shaped receiver, the polymer spinning solution will form a spinning jet under the stretching of the electric field force, which will evaporate through the solvent to form “like‐spider web” micro/nanofibers. When two sets of micro/nanofibers gather at the edge of the rotary funnel receiver, the fiber mesh will be wound into yarn. Subsequently, the oriented LCE long yarn soft actuator was stably manufactured via a roller drafting and UV crosslinking strategy. The preparation process, morphology, and structural changes of electrospun nanofiber yarns are shown in Figure 1B–D. Figure 1D presented that the preparation of electrospun liquid crystal elastomer fiber yarn is mainly divided into three processes. First, an addition reaction between liquid crystal monomers (RM257), AuNRs monomers and chain extender (EDDET) was carried out and liquid crystal oligopoly fibers network was prepared by electrostatic spinning technique. Second, the liquid crystal fiber mesh is rotated and twisted to prepare LCE fiber yarns with irregular orientation. At this time, the connection points in the fiber yarns are cross‐linked by UV to form relatively stable binding points. Finally, fiber yarns were subjected to a drafting process and UV cross‐linking and sizing strategy to prepare orientationally aligned electrospun AuNRs@LCE yarn soft actuator. During the UV cross‐linking process, the carbon–carbon double bonds of liquid crystal monomers and AuNRs monomers undergo addition reaction, resulting in the formation of long‐chain macromolecules polymer.

**Figure 2:**
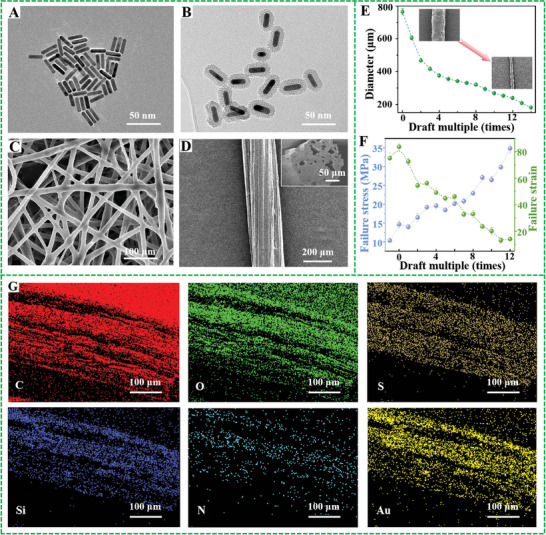
A,B) The TEM image of functionalized AuNRs before and after modification; C) the SEM image of electrospun AuNRs@LCE micro/nanofibers; D) SEM images of AuNRs@LCE yarn (The illustration shows the longitudinal sections morphology); E,F) relationship between draft multiple and diameter, mechanical properties of electrospun AuNRs@LCE yarn; G) EDS elemental energy spectra of AuNRs@LCE yarn.

As shown in **Figure** **2**A, numerous AuNRs with uniform size and stable aspect ratio were prepared via using the seed growth method. And TEM images can clearly indicate that the size distribution of the synthesized AuNRs is very uniform, ranging from 16 to 28 nm, with an average length of about 20 nm and an average aspect ratio of 4 (Figure S1B–D, Supporting Information). After surface functionalization modification, a uniform mesoporous silicon layer can be clearly observed on the surface of photopolymerizable AuNRs molecules (Figure 2B; Figure S1C, Supporting Information). The successful functionalization of AuNRs monomers were further verified through fourier transform infrared (FTIR) spectroscopy (Figure S2, Supporting Information).^[^ ^35^ ^]^ Results clearly indicated that grafting of amino group (─NH_2_) onto the silica@gold nanorods (SiO_2_@AuNRs) surface and subsequent formation of the covalently‐bonded AuNRs nanomonomer.^[^ ^33^ ^]^ Then, the optical properties of the prepared AuNRs were analyzed by UV‐visible spectrum analyzer (UV–vis), and the results are shown in Figure S3 (Supporting Information). The synthesized AuNRs have two bands: a strong long‐wave band at 809 nm due to longitudinal electron oscillations, and a weak short‐wave band near 510 nm due to transverse electron oscillations. Compared with the AuNRs, the UV–vis of the prepared polymerizable AuNRs monomer showed a strong absorption band at 812 nm and a weak absorption band at 513 nm (Figure S3B, Supporting Information). The results exhibited that the surface modification technique has little effect on UV–vis absorption of AuNRs. After high‐voltage electrospinning technology, a biomimetic “like‐spider web” disordered AuNRs@LCE micro/nanofibers network is first formed on the surface of the rotating receiver, with a uniform fiber diameter ranging from 24 to 30 µm, good fiber morphology, and obvious cross bonding points and networks structure formed between different fibers due to incomplete solvent evaporation (Figure 2C; Figure S4A, Supporting Information). When stimulated by external light, the uniformly distributed AuNRs could produce stable photothermal effects inside the fabricated yarn. Subsequently, the scalable AuNRs@LCE yarn was stable manufactured through stretching and UV crosslinking strategies (Video S1, Supporting Information).

The FTIR of polydomain AuNRs@LCE nanofiber yarn demonstrated that the peak of thiol group disappeared, and the strength of C═C bond weakened, suggesting that the thiol group and double bond had Michael addition reaction. After drawing and UV light crosslinking treatment, the spectrum of monodomain AuNRs@LCE nanofiber yarn reveals that the peak of C═C bond disappears, proving that the C═C bond in the yarn actuator had an addition reaction (Figure S4B, Supporting Information).^[^ ^32^ ^]^ Figure 2D demonstrated that the diameter of the yarn is 239±11 µm under the condition of being stretched 12 times, while the electrospun micro/nanofibers inside the yarn exhibited a good orientation distribution. From the longitudinal sections view of the yarn, it can be observed that liquid crystal yarn is composed of numerous micro/nanofibers, and the fiber network undergoes partial crosslinking during the stretching and UV light crosslinking process, resulting in tight bonding between micro/nanofibers. The above results clearly confirmed the successful preparation of AuNRs@LCE nanofiber yarns actuator using a combination of electrospinning technology and two‐step cross‐linking strategy.

We also noticed that the diameter of electrospun AuNRs@LCE yarns gradually decreases with the increase of the drawing multiple (Figure 2E; Figure S5, Supporting Information). Meantime, Figure S5 (Supporting Information) demonstrated that the diameter of both the yarn and internal fibers gradually decreases with the increase of the drafting multiple, and the liquid crystal elements inside the fibers gradually change to be arranged along the stretching direction. In addition, the alignment of different fibers in the yarn along the stretching direction also gradually increase. To evaluate the practical application prospects of the obtained yarns, the mechanical properties of electrospun yarn with different draft multiple have been deeply explored. As shown in Figure 2F, as the drafting ratio gradually increased, the failure strain of the electrospun yarn actuator gradually decreased, while the failure stress values raised. The energy dispersive spectrometer exhibited that pure LCE micro/nanofibers yarn only contains three elements of C, O and S (Figure S6, Supporting Information).^[^ ^22^, ^36^ ^]^ However, the designed AuNRs@LCE yarns soft actuator, due to the polymerization of AuNRs monomers on the liquid crystal polymer molecular chain through silane coupling method, not only contained C, O and S elements, but also uniformly distribute Si, N and Au elements on the yarn soft actuator. This phenomenon further confirmed the AuNRs monomer modified by silane coupling is uniformly polymerized and stable loaded inside the electrospun AuNRs@LCE yarn (Figure 2G; Figure S7, Supporting Information).

**Figure 3:**
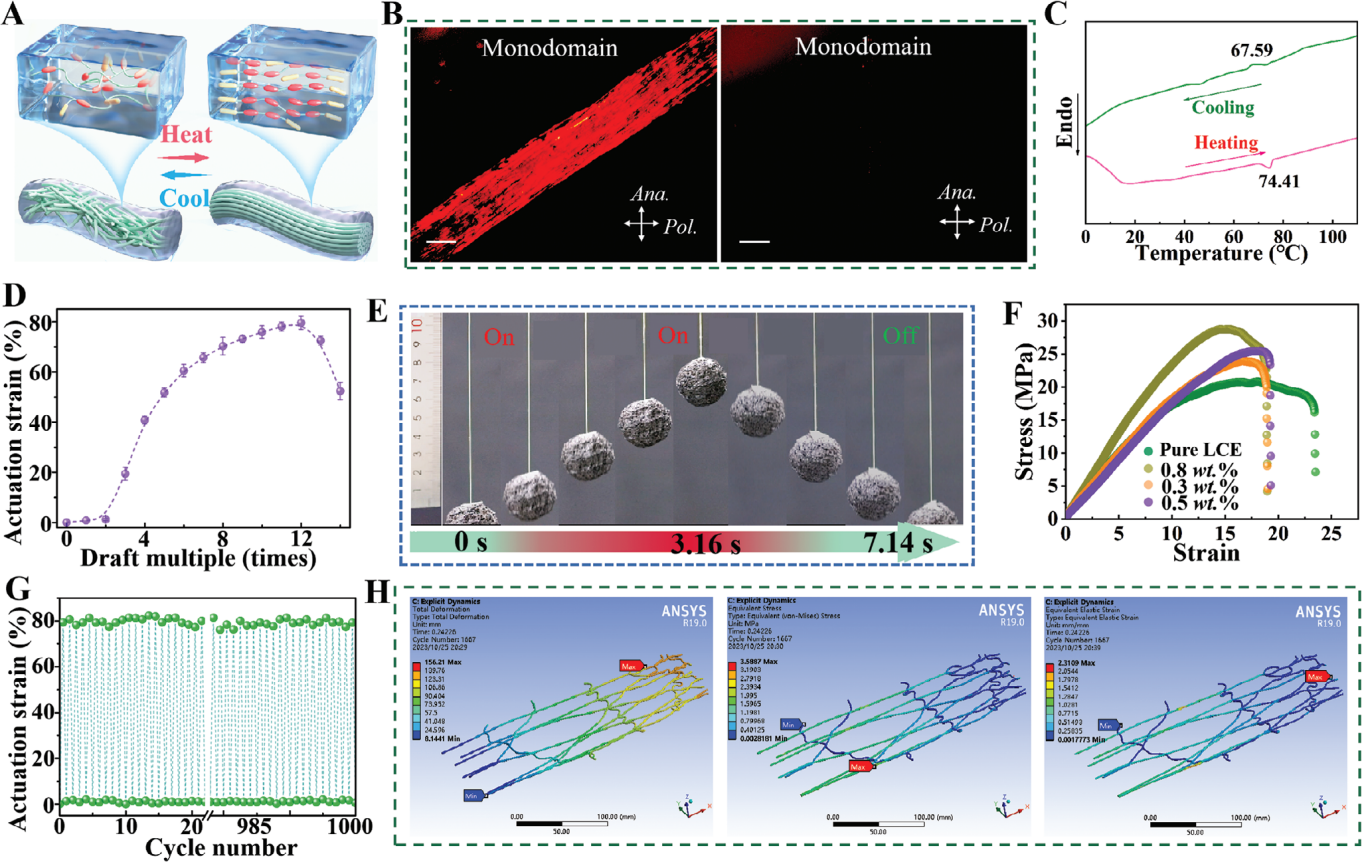
Thermal stimulation response super‐contraction mechanism and mechanical performances of AuNRs@LCE yarns. A) Diagram of reversible thermal actuation of the designed yarns with different alignment domains network at 25 and 120 °C, respectively. B) Polarization microscope (POM) images of AuNRs@LCE yarns viewed at two different angles with respect to the analyzer. C) The DSC curve of as‐prepared yarns. D) Relationship between draft multiple and actuate rate of electrospun AuNRs@LCE yarn. E) Physical photos images of the prepared yarn lifting a round ball (400 mg). F) The stress–strain curve of different as‐prepared nanofiber yarns. G) Exhibits the thermal drive reusability of electrospun yarn soft actuator. H) The stress and deformation during the stretching process of electrospun nanofiber membrane simulated by finite element method. Scale bars, 100 µm.

In order to elucidate the internal actuation mechanism of electrospun AuNRs@LCE yarn, the obtained yarns were systematically characterized and their actuation properties were analyzed in this study. **Figure** **3**A exhibited that as‐prepared yarn is composed of numerous electrospun micro/nanofibers, which presented spontaneous and reversible shrinkage during heating and expansion along the fiber axis during cooling. There are two main reasons for this significant thermal reversible deformation: First, similar to the contraction of a single fiber in a spider web, numerous mesogenic units in the polymer network of each micro/nanofiber will undergo shrinkage deformation due to phase transitions stimulated by external temperature. Second, the fabricated electrospun AuNRs@LCE yarn contains numerous robust fiber networks formed by different micro/nanofibers, and this fiber network structure distribution is similar to the elastic fiber interwoven web formed by spider spinning. Due to the reversible deformation of a single micro/nanofiber, the above robust fiber network formed within the electrospun yarn undergoes stable shape deformation, further enhancing the actuation performance of the obtained yarn. We assumed that this hierarchical structure formed between liquid crystal molecules, single fibers, fiber networks, and yarns achieves the super reversible deformation effect of electrospun yarns. The polydomain AuNRs@LCE yarn with isotropic orientation was manufactured by rotating electrospinning technique, and then the monodomain yarn was prepared through drafting and UV crosslinking method. We noted that the polydomain AuNRs@LCE yarn presented no difference in the brightness when observed at 45° or 90° with respect to the analyzer because the nematic phase domains are randomly distributed (Figure S8, Supporting Information). When the polydomain yarn actuator was stretched, numerous micro/nanofibers within the yarn exhibited oriented arrangement state, and the liquid crystal mesogens displayed a nematic arrangement state.^[^ ^22^ ^]^ At this point, it can be clearly observed in Figure 3B and Video S2 (Supporting Information) that the yarn actuator presented significant differences in brightness at 45° and 90° with respect to the analyzer. In addition, differential scanning calorimetry image of AuNRs@LCE yarn depicted that the nematic isotropic phase transition and glass transition temperature were about 74.4 and 13.8°C, respectively (Figure 3C).

As can be seen from Figure 3D, the actuation performances of the yarn gradually enhanced with the gradual increase of the draft multiple. When the as‐prepared draft ratio reached 12 times, the yarn soft actuator demonstrated super‐contractile performances, and its actuation stain up to 81.2%. When the draft ratio was further increased, the AuNRs@LCE yarn driving performance decreased. The main reason for this phenomenon was that with the increase of the drafting ratio, the degree of nematic alignment of the liquid crystal mesogens in the fiber was gradually raised, and the degree of orientation of many micro/nanofibers in the yarn along the stretching direction is increased, resulting in the actuation strain of the yarn is gradually increased. However, the part of the fiber network and some fibers were destroyed by continuing to increase the drawing ratio, resulting in the designed yarn driving rate is reduced. Based on the requirements for yarn weaving performances and actuating performances, this work identified nanofiber yarns with a draft ratio of 12 times as further research objects. Figure 3E and Video S3 (Supporting Information) displayed that physical and infrared image of the large reversible drive deformation (≈ 81%) achieved by the yarn soft actuator under 3.16 s of thermal stimulation. The infrared image in the illustration of Figure S9 (Supporting Information) further proved that the generated AuNRs@LCE yarn actuator was deformed due to heating.

In addition, to explore the practical application capabilities of yarn actuator, we determined and analyzed the mechanical performances and thermal actuation reusability, as shown in Figure 3F,G and Figure S10 (Supporting Information). The stress–strain curves indicated that the breaking strength (23.92–28.74 MPa) and Young's modulus (1.26–1.54 MPa) of the LCE yarn actuator with different AuNRs contents was higher than that of pure LCE yarn (20.77 MPa, 0.88 MPa). However, the opposite phenomenon occurs in the fracture strain of yarn actuator. The reason was that functionalized AuNRs undergo addition reactions with the mercapto groups of the chain extender or the carbon–carbon double bonds of the end groups of the liquid crystal monomers, which leads to a polymerization process within the molecular chain of the LCE. Noted that numerous chemical covalent bound AuNRs were more difficult to move during the stretching process than liquid crystal molecules, resulting in an increase in the breaking strength of the fibers. At the same time, the incorporation of inorganic AuNRs enhanced the rigidity of the fiber yarns and affected the structure of the molecular chain arrangement of the liquid crystal network, making it more difficult for the polymer molecular chains to move during stretching, thereby reducing the fracture strain of the LCE. Additionally, the reuse and thermal stability performances images in the illustration of Figure 3G and Figure S11 (Supporting Information) exhibit that the obtained AuNRs@LCE yarn actuator possessed stable driving characteristics and good thermal stability after 1000 times of reuse, which could meet daily environment and operational requirement.

In order to reveal the tensile deformation mechanism of electrospun AuNRs@LCE based micro‐nano fiber network, a simple random fibers network was designed and analyzed by finite element analysis method (FEA), as shown in Figure S12 and Video S4 (Supporting Information). The FEA results (the total strain‐time, equivalent strain‐time, equivalent stress‐time nephograms) of the AuNRs@LCE fibers network with 100% strain are shown in Figure 3H. According to the total strain distribution and equivalent strain distribution of the designed AuNRs@LCE fibers network, the deformation of the single fiber gradually increases with the gradual increase of the tensile strength, and the fiber mesh gradually extends and deforms along the stretching direction, and the width of the fiber mesh gradually decreases. As the difficulty of fiber stretching deformation gradually increases, the stress acting on a single fiber gradually disperses in the fiber network, causing the fiber network to further elongate and deform along the stretching direction. In addition, the equivalent stress‐time distribution nephograms of the fibers network demonstrates that the stress borne by fibers with the same direction of force distribution is significantly greater than that of fibers with different directions. The main reason for this phenomenon is that fibers with the same direction of force will transmit more load during stretching.^[^ ^37^, ^38^ ^]^ The above FEA results indicate that the fibers in the electrospun AuNRs@LCE yarn not only undergo orientation deformation, but also the different fibers network in the yarn shows orientation structure in the stretching direction before the as‐prepared yarn is crosslinked by the UV light.

**Figure 4:**
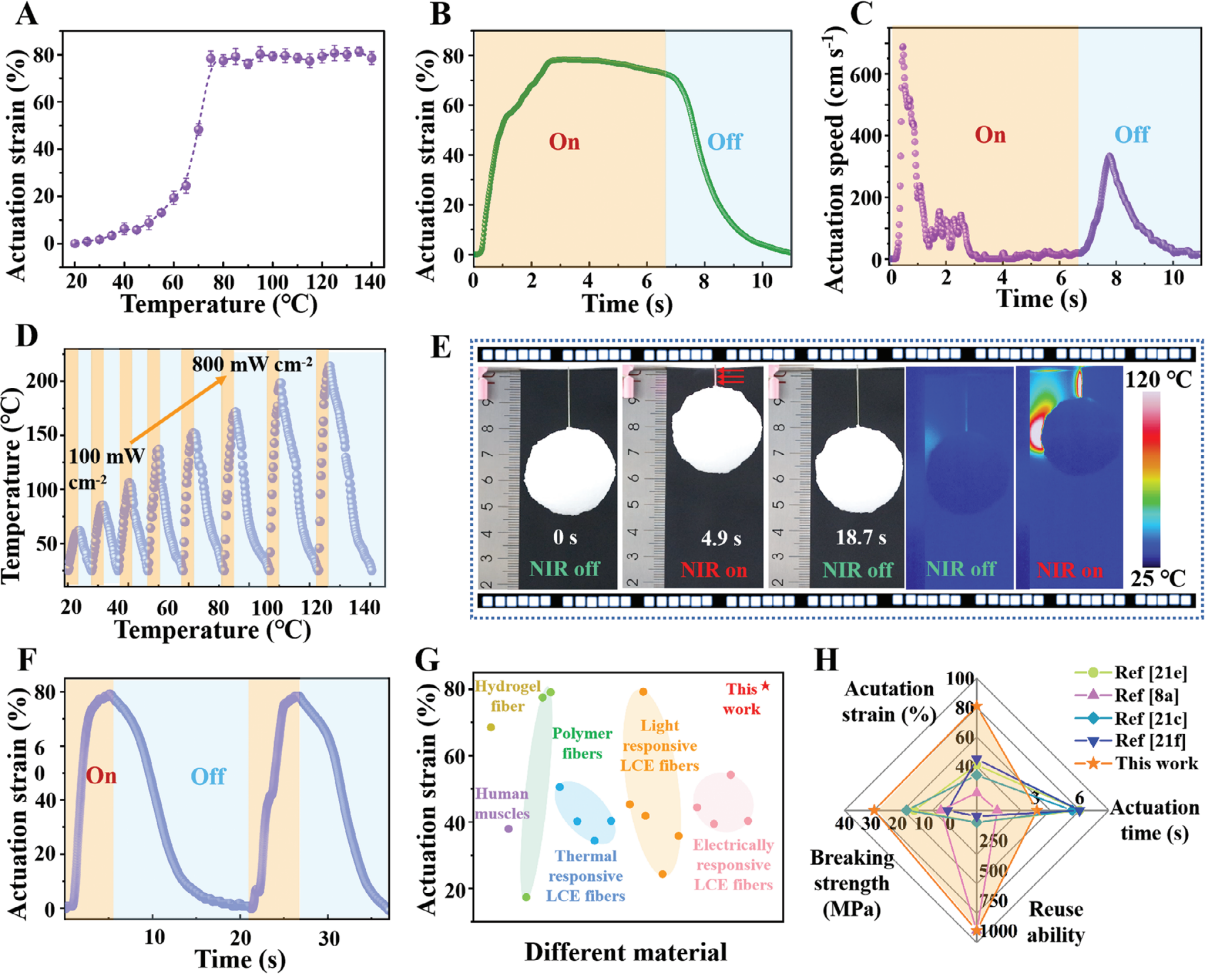
Light/thermal actuation performances of AuNRs@LCE active yarn soft actuator. A) Relation curve between the actuate strain of yarn actuator and temperature. B,C) Relationship between actuation strain, speed and time, respectively. D) The photo‐thermal temperature of the yarn actuator under different light intensities. E) Physical photos and infrared images of as‐prepared active yarn actuator lifting a round ball (600 mg). F) Relationship between the NIR light‐driven strain and time. G) A comparison diagram of the actuation strain of the designed yarn actuator with reported literatures. H) A radar diagram of the actuated performances of the AuNRs@LCE yarn actuator with reported LCE based fibers actuators.

Subsequently, the thermal actuation performances of yarns actuators were further analyzed through fitting calculations of the thermal stimulus response actuation process. It can be seen that the obtained AuNRs@LCE yarn actuator beginning to actuate strain at about 40°C and decreasing to 79.5% of its initial length at around 75°C with the gradual increase of ambient temperature from **Figure** **4**A. In addition, Figure 4B revealed that the LCE based yarn soft actuator could achieved stable reversible actuate deformation under the condition of temperature stimulation, and the actuate deformation strain is as high as nearly 81%, which is significantly higher than the literature reports (Table S1, Supporting Information).^[^ ^14^, ^39^, ^40^, ^41^ ^]^ Moreover, the change curve of actuation velocity and acceleration displayed that when the stimulation temperature was 100°C, the maximum actuation speed and acceleration of electrospun AuNRs@LCE yarns soft actuator could reach 688 cm s^−1^ and 6260 cm s^−2^, respectively (Figure 4C; Figure S13A, Supporting Information). Surprisingly, the generated yarn actuator can lift up to 1500 times its own weight, demonstrating excellent weight lifting ability. And noted that when it is loaded with 1000 times itself weight, its actuation strains can still reach over 55% (Figure S13B, Supporting Information). These results further indicated that the fabricated electrospun yarns actuator has excellent thermal actuate capability.

To endow LCE materials with photo‐thermal actuate ability, this study introduced polymerizable AuNRs with excellent photothermal conversion performances into the LCE polymer molecular chain. Figure 4D–F and Figure S14 (Supporting Information) demonstrated that the photo‐thermal effect and light‐driven performances of the fabricated AuNRs@LCE yarn soft actuator under the condition of near‐infrared (NIR) light stimulation. As illustrated in Figure S14A (Supporting Information), as the near‐infrared (500 mW cm^−2^) irradiation time gradually prolonged, the photothermal temperature generated by the active yarn soft actuator gradually increased. Furthermore, the higher the amount of AuNRs added in the active yarn, the higher the photothermal temperature generated on the surface. When the fabricated AuNRs@LCE yarn actuator is stimulated by NIR light with different light intensities (100–800 mW cm^−2^), the produced highest photo‐thermal temperature and the heating‐rate of the yarn actuator gradually raised with the increased in light intensity (Figure 4D). Nevertheless, the actuation strain of the obtained AuNRs@LCE yarn actuator first increased with the increase of light intensity and then gradually tend to balance (Figure S14B, Supporting Information).

We noted that the maximum reversible deformation of the obtained AuNRs@LCE yarn actuator driven by NIR light could reach 79.8% within 4.9 s under 500 mW cm^−2^ of NIR light stimulation (Figure 4E,F; Video S5, Supporting Information). The infrared image in the illustration of Figure S15 (Supporting Information) further confirmed that the generated yarn actuator was deformed because of the photothermal conversion. Meanwhile, the actuation speed and acceleration of the NIR light were calculated to reach 74 cm s^−1^ and 233 cm s^−2^, respectively (Figure S16A,B, Supporting Information). The AuNRs@LCE yarn actuator was also shown to have a stable NIR light‐driven deformation capacity after 1000 repetitions (Figure S16C, Supporting Information).

Compared with the relevant reported literature, most of the light, thermal, and electrical‐driven strain rates of LCEs based fiber actuators were below 50%, and the actuation strain of hydrogel actuator and human muscle are basically lower than 70%.^[^ ^3^, ^15^, ^18^, ^21^, ^29^, ^30^, ^39^, ^40^, ^41^, ^42^, ^43^, ^44^, ^45^, ^46^, ^47^ ^]^ Only a few shape memory polymers and LCE based actuators can achieve an actuation rate of 80%, but the complex synthesis process and cumbersome preparation process of this polymer limited its further large‐scale application^[^ ^7^, ^48^ ^]^ (Figure 4G,H; Table S1, Supporting Information). Surprisingly, the actuation strain achieved in our work is two times larger than most reported conventional light‐driven fiber actuators, the designed scalable AuNRs@LCE yarn actuator presented better super‐contractile performances, and the yarn manufacturing process is convenient and efficient and easy to large‐scale production (Figure 4G). Figure 4H highlights except for the actuation time that is comparable to the previously reported values, the other parameters are noticeably higher than that of existing different LCE fiber actuators,^[^ ^21^, ^44^, ^46^, ^47^ ^]^ these characteristics (e.g., super‐contractile performances and reusability) place our designed biomimetic “like‐spider web” structure of the AuNRs@LCE active yarn actuator to cover previously inaccessible regions of the performance comparison charts of fiber LCE actuators. Above results demonstrated that the biomimetic hierarchical structure of the electrospun active yarn not only endows the actuator with photo/thermal large reversible shape‐morphing characteristics, but also possesses excellent structural stability, making it demonstrate good application prospects in practical applications.

**Figure 5:**
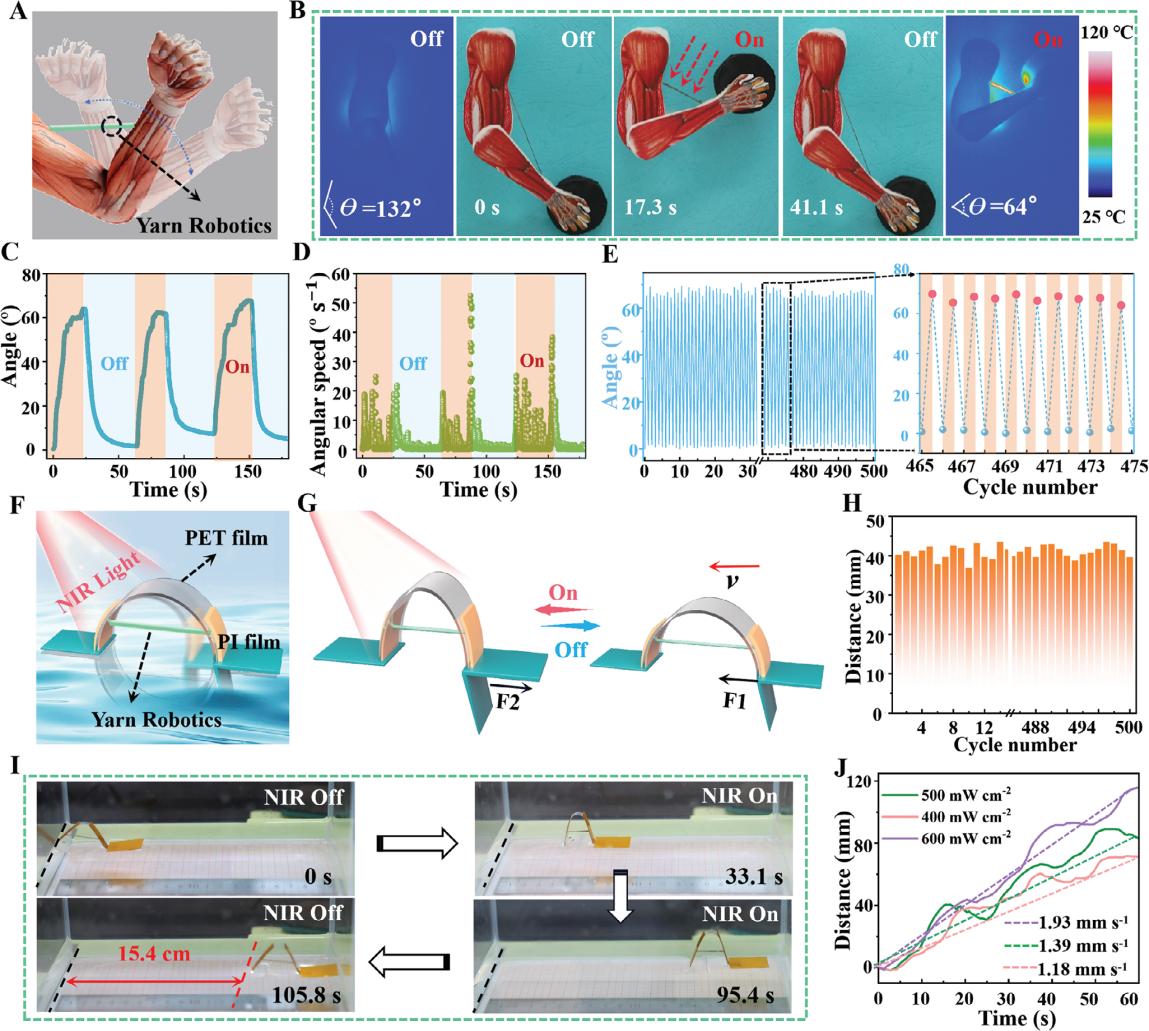
A) Schematic of the NIR light‐driven artificial arm model is composed of the AuNRs@LCE active yarn simulating biceps. B) The physical and infrared images of arm bending and restoration under NIR light stimulation. C–E) Elbow bending angle, angular bending velocity and repeatability of designed artificial arm. F) Schematic of a micro swimmer is mainly composed of the active yarn. G–I) Schematic of movement, reusability, and physical movement of micro swimmer under NIR light stimulation. J) The relationship between the moving distance and the illumination intensity.

To further expand the practical application prospects of yarn soft actuator, we have designed and constructed light‐controlled super‐contractile artificial muscle, micro swimmers and hemostatic bandages based on the obtained active yarn. Moreover, photo‐thermal actuation process of three scenarios has systematically analyzed. Noted that mammalian muscle tissue is composed of numerous muscle fibers that can be rapidly reversible and highly controllable.^[^ ^49^ ^]^ Herein, the active yarn actuator was simulated as the human biceps femoris and a super‐contractile artificial muscle model was manufactured, as shown in **Figure** **5**A. It can be found that when the AuNRs@LCE yarn soft actuator (0.002 g) is irradiated with NIR light, it generated nearly 80% of large reversible deformation, enabling in the arm's bend and lift 3 g weight in the Figure 5B,C and Video S6 (Supporting Information). Meanwhile, Figure S17 (Supporting Information) depicted that the active yarn was heated by numerous AuNRs photothermal conversion effect under continuous stimulation of 500 mW cm^−2^ NIR light. Subsequently, the generated yarn soft actuator underwent reversible contraction deformation, resulting in a reversible bend of up to 68° in the constructed artificial muscle model. Figure 5D and Figure S18 (Supporting Information) demonstrated that the maximum angular velocity and acceleration of the designed artificial muscle were 52.4 ° s^−1^ and 473.2 ° s^−2^, respectively. These results are significantly higher than reported literature.^[^ ^18^, ^32^, ^47^ ^]^ In addition, the artificial muscle remained stable ability to deform after 500 repetitions, indicating satisfactory reuse characteristics (Figure 5E).

As shown in Figure 5F, a flexible micro swimmer consisting of polyimide tape (with curved ends), polyethylene terephthalate (PET) film, and AuNRs@LCE yarn was designed and prepared. When the AuNRs@LCE active yarn was exposed to the NIR light, due to the large contact area and strong adhesion force between the PET film at the front end of the micro swimmer and the water surface, the contraction force (**F1**) generated by the yarn actuator causes the PET film to bend and fold, thereby the PET film at the rear end moved Forward. After the NIR light stimulation is removed, the yarn elongation returns to the initial length, the folded film gradually opens and returns to its original shape. Meantime, PET film folded at the rear end pushed the water backward, which could generate a thrust force (**F2**) to make the entire body to move forward on the surface of water (Figure 5G; Video S7, Supporting Information). Thanks to the super actuation strain and excellent light‐driven stability of the active yarn, our designed micro swimmer can quickly move 4.1 cm on the water surface per stimulation cycle (600 mW cm^−2^), and still maintains stable mobility after 500 cycles (Figure 5H; Figure S19, Supporting Information). In addition, this study has shown that the micro swimmer can moved at speeds of 1.2‐, 1.4‐, and 1.9 mm s^−1^ under different light intensities (400–600 mW cm^−2^). Under the continuous stimulation of 600 mW cm^−2^ of NIR light, it can continuously travel 15.4 cm within 105.8 s, which is significantly higher than the results reported in the literature (Figure 5I,J).^[^ ^21^, ^33^ ^]^

**Figure 6:**
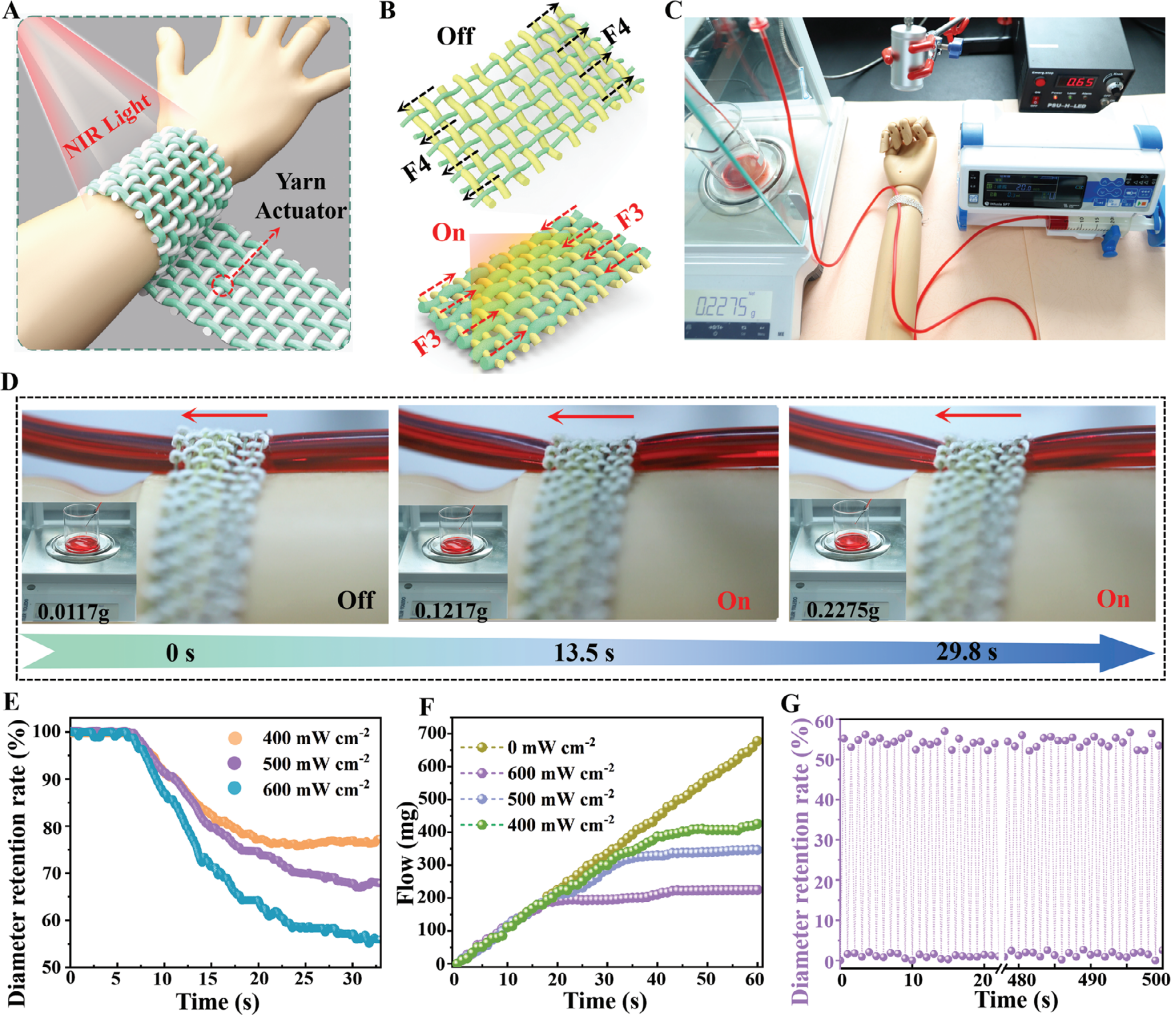
A,B) Schematic diagram and reversible deformation mechanism of a light‐controlled hemostatic bandage, respectively. C) Physical image of using a light‐controlled hemostatic bandage to slow down fluid flow on a simulated arm. D) The physical image of the light‐controlled hemostatic bandage tightening the infusion tube, with the illustration showing the real‐time quality of the flowing liquid. E–G) The deformation rate of the infusion tube, the weight of the flowing liquid, and the reusability of the light‐controlled hemostatic bandage, respectively.

Due to the excellent photothermal actuation and weaving characteristics of the active yarn, we have adopted textile processing technology to manufacturing a novel light‐controlled hemostatic bandage. The model and actuation process of the hemostatic bandage are shown in **Figure** **6**A,B. The AuNRs@LCE actuator is used as warp yarn and cotton as weft yarn, and the intelligent bandage is woven according to the plain weave fabric structure. Laser transmitter, injection pump, plastic infusion tube, self‐made light‐controlled hemostatic bandage and artificial arm model were used to simulate the action process of the photocontrolled hemostatic bandage, as shown in Figure 6C. When NIR light simulation, the active yarn in the bandage is heated and contracted due to the photothermal effect (**F3** as photothermal contraction force, **F4** as deformation recovery force), tightening the infusion tube on the arm, thereby reducing the flow rate of the infusion tube and causing a decrease in the weight gain rate of the liquid flow on the left electronic balance (Video S8, Supporting Information).

As can be seen from the physical images in Figure 6D and the infrared images in Figure S20 (Supporting Information), with the extension of NIR light stimulation time, the designed light‐controlled hemostatic bandage gradually heats up and shrink, which then compresses and deforms the infusion pipeline simulating artificial blood vessels and reduces the liquid flow rate. Results exhibited that the deformation of infusion pipeline could reach up to 44.2% with the gradual increase of light intensity (Figure 6E). Moreover, the deformation speed and acceleration of the infusion tube could reach 7.8 mm s^−1^ and 74.4 mm s^−2^, respectively (Figure S21, Supporting Information). It also can be observed that after continuous NIR irradiation (600 mW cm^−2^), the active yarn in the bandage continuously shrinks due to photothermal temperature in Figure 6F. Subsequently, the infusion tube on the arm model was then tightened and the flow of the infusion tube was reduced to a maximum of 453 mg in 1 min. Moreover, the research suggested that the constructed smart hemostatic bandage still presents excellent light‐controlled shrinkage characteristics after 500 times of repeated use, which is attributed to the long‐term stable light‐driven characteristics of the as‐prepared active yarn (Figure 6G). Although the light‐controlled hemostatic bandage is limited by factors such as high actuation temperatures and an insufficient light‐driven response speed in practical applications, as a concept that combines smart actuation with healthcare, it will give smart wearables and medical textiles a new way to be designed in the future.

## 3 Conclusion

In summary, inspired by the structure and function of spider webs, we have demonstrated a facile strategy to design and fabricate novel scalable AuNRs@LCE active yarn with super‐contractile capability and stable actuation characteristics through a combination of electrospinning technique and intramolecular polymerization method. We have systematically characterized electrospun active yarn and comprehensively analyzed its photo/thermal actuation performances. Results revealed that the photopolymerizable AuNRs were crosslinked evenly inside the LCE molecular chain, and the active yarn showed long‐term stable light absorption and photothermal actuation performances. Thanks to the advantages of the hierarchical structure characteristic of bionic “like‐spider webs”, the electrospun fiber network structure in the AuNRs@LCE active yarn displayed super‐contractile actuation strain (≈81%) and stable and durable shape‐morphing characteristics compared with the current LCE fiber actuators. Based on flexible and weavability properties of the prepared active yarn and its long‐term photo‐thermal actuation capacity, we designed and constructed the light‐controlled super‐contractile artificial muscle, micro swimmer and smart hemostatic bandage. Note that the designed light‐driven micro swimmer could move quickly on the surface of the water, and the smart bandage could effectively achieve the hemostatic effect under light stimulation. This work opens up a new avenue to integrate and development of soft actuator and smart wearable devices based on the fabricated LCE active yarn.

## 4 Experimental Section

### Materials

The experimental materials, reagents, and monomer synthesis process required for this study can be found in Supporting Information.

### Fabrication of the AuNRs@LCE Active Yarn

In this experiment, the AuNRs@LCE active yarn was manufactured through using molecular synthesis and electrospinning method. During the electrospinning process, dual spinnerets were maintained at positive (10 kV) and negative potentials (−8 kV) and a uniform flow rate of 1.2 ml h^−1^ using an infusion pump was applied to polymeric solutions taken in a syringe at a concentration of 24 *wt*.%, yielding active yarn with AuNRs@LCE micro/nanofibers. Meanwhile, the AuNRs@LCE fiber was irradiated by ultraviolet lamp with wavelength of 365 nm during the spinning and drafting process, so that the second step of cross‐linking reaction occurs in the micro/nanofibers. Finally, the scalable AuNRs@LCE active yarn with high orientation and uniform diameter are continuous manufactured.

### Finite Element Analysis (FEA)

FEA was performed using Ansys Workbench software (ANSYS STUDENTS 2019). The electrospun AuNRs@LCE micro/nanofibers were modeled as a hyperelastic material. The micro/nanofiber was modeled as an elastic isotropic material with an elastic modulus of 2.8 × 10^6^ MPa and Poisson's ratio of 0.42.^[^ ^37^, ^50^ ^]^ Considering the actual distribution of fibers in LCE based yarn actuator, the contact relationship between micro/nanofibers was set for stable bonding. The model contained 6399 nodes and 1126 elements after automatic meshing.

### Actuation Performances of AuNRs@LCE Active Yarn

To determine the light/thermal actuation performances of the designed active yarn, a thermal drying gun and a 808 nm NIR light laser emitter was applied to the surface of the active yarn, respectively. Then, the light/thermal actuation strain and temperature changes of the AuNRs@LCE yarn actuators were recorded by a Canon camera and an infrared imaging system, and actuating deformation speed and acceleration of the yarn actuator were analyzed through using Kinovea software. Finally, the light/thermal super‐contractile capability of the AuNRs@LCE yarn actuator was systematically investigated.

### Fabrication of the Light‐Controlled Artificial Muscle, Micro Swimmer, and Smart Hemostatic Bandage

First, an artificial muscle model based on the AuNRs@LCE active yarn was created. Meantime, the contraction and recovery of light‐controlled artificial muscle yarn was recorded by a Canon camera. Second, the light‐driven micro swimmer was composed of a bending structure made from polyethylene terephthalate film, polyimide tape (with curved ends), and AuNRs@LCE yarn. Finally, a smart hemostatic bandage was designed with the plain weave fabric structure by weaving the as‐prepared AuNRs@LCE actuator as warp yarn and cotton as weft yarn.

## Conflict of Interest

The authors declare no conflict of interest.

## Supporting information

Supporting Information

Supplemental Video 1

Supplemental Video 2

Supplemental Video 3

Supplemental Video 4

Supplemental Video 5

Supplemental Video 6

Supplemental Video 7

Supplemental Video 8

## Data Availability

The data that support the findings of this study are available from the corresponding author upon reasonable request.
