# Supplementary material for: Novel Biomimetic “Spider Web” Robust, Super‐Contractile Liquid Crystal Elastomer Active Yarn Soft Actuator
Source: Adv Sci (Weinh). 2024 Feb 28;11(17):2400557. doi: 10.1002/advs.202400557 (PMC11077665; doi:10.1002/advs.202400557)
Supplement: Supplementary file 1 — Supporting Information [file ADVS-11-2400557-s009.pdf]

ADVANCED SCIENCE
Open Access

Supporting Information

for *Adv. Sci.*, DOI 10.1002/advs.202400557

Novel Biomimetic "Spider Web" Robust, Super-Contractile Liquid Crystal Elastomer Active Yarn Soft Actuator

Dingsheng Wu, Xin Li, Yuxin Zhang, Xinyue Cheng, Zhiwen Long, Lingyun Ren, Xin Xia, Qingqing Wang, Jie Li, Pengfei Lv, Quan Feng* and Qufu Wei**

Supporting Information for

Novel Biomimetic "Spider Web" Super-Contractile Liquid Crystal Elastomer Active Yarn Soft Actuator

Dingsheng Wu,[1,2] Xin Li,[1] Yuxin Zhang,[1] Xinyue Cheng,[1] Zhiwen Long,[1] Lingyun Ren,[1] Xin Xia,[3] Qingqing Wang,[1] Jie Li,[1] Pengfei Lv,[,1] Quan Feng,*[,2] and Qufu Wei*[,1]*

[1] Key Laboratory of Eco-Textiles, Ministry of Education, Jiangnan University, Jiangsu 214122, China.
[2] Key Laboratory of Textile Fabrics, College of Textiles and Clothing, Anhui Polytechnic University, Anhui 241000, China.
[3]College of Textile and Clothing, Xinjiang University, Xinjiang, Urumchi 830046, China

*E-mail: qfwei@jiangnan.edu.cn (Q.W.); pengfeilv@jiangnan.edu.cn (P.L.); fengquan@ahpu.edu.cn (Q.F.)

Contents

1. Experimental Procedures

1.1 Materials

1.2 Synthesis

1.3 Measurements and Characterization

1.4 Actuation Performances of AuNRs@LCE Yarn Soft Actuator

1.5 Fabrication of Light-Controlled Artificial Muscle, Micro Swimmer and Smart Hemostatic Bandage

2. Supplementary Figures and Table

3. Captions of Supplementary Video

1. Experimental Procedures

1.1. Materials

Chloroauric acid ($HAuCl_4$), hexadecyl trimethyl ammonium bromide (CTAB), sodium borohydride ($NaBH_4$), silver nitrate ($AgNO_3$), hydroquinone, and dipropylamine (DPA) were purchased from Sinopharm Chemical Reagent Co., Ltd. (Beijing, China). 2-hydroxy-4'-(2-hydroxyethoxy)-2-methylpropiophenone (HHMP), hydrochloric acid (HCl, 36 *wt*% in H_2O), 3,6-dioxa-1,8-octanedithiol (EDDET), dichloromethane (CH_2Cl_2), sodium hydroxide (NaOH), tetraethyl orthosilicate (TEOS), (3-aminopropyl) triethoxysilane (APTES), tetrahydrofuran (THF), and 1-Ethyl-3-(3-dimethylaminopropyl) carbodiimide hydrochloride (EDC·HCl) were obtained from Aladdin Reagent Co., Ltd. (Shanghai, China). 1,4-bis- [4-(3-acryloyloxypropyloxy) benzoyloxy]-2-methylbenzene (RM257), 4-((6-(acryloyloxy) hexyloxy) benzoic acid (6OBA) were received from Shijiazhuang Yesheng Chemical Technology Co. Ltd. (Hebei, China). All materials were used without purification.

1.2. Synthesis

Synthesis of AuNRs Nanomonomers: Numerous gold nanorods **(**AuNRs) were synthesized with hydroquinone-based seedless growth method. It is worth noting that the volume of $AgNO_3$ determines the morphology of the MiniGNRs. The AuNRs whose longitudinal LSPR peaks centered at 809 nm were used by adding $AgNO_3$ (aq, 0.01 M) with volumes of 120 µL in this study. Subsequently, silica coating was carried out using a modified Stöber method [1]. CTAB and TEOS methanol solution were added to AuNRs solution (*p*H, 10.6) for 20 h. Then the solution was cleaned by centrifugation at 12,000 rpm for 20 min twice. Then, APTES was added to AuNRs@SiO_2 ethanol solution. The mixture was then heated to 70 ℃ for 48 h under continuously stirring and nitrogen atmosphere, followed by extensively washing with ethanol by centrifugation at 10,000 rpm for 10 min twice. Finally, 6OBA and EDC·HCl were added to the THF solution of AuNRs@SiO_2-NH_2 nanoparticles and reacted for 2 days. Subsequently, the mixture was cleaned by centrifugation at 10,000 rpm for 20 min. The resultant nanoparticles were dried under high vacuum to obtain AuNRs nanomonomer.

Synthesis of the AuNRs@LCE Electrospinning Solution: Liquid crystal monomer (RM257) and chain extender (EDDET) were dissolved in CH_2Cl_2 solution, then the functionalized AuNRs monomers were uniformly dispersed in the composite solution using ultrasonic

dispersion technology (the molar ratio of the AuNRs monomers, RM257, and EDDET was 1:11000:10000). Subsequently, the catalyst DPA as a catalyst was added dropwise into the above homogeneous solution to cause the system undergo a Michael addition reaction. (The carbon-carbon double bonds within the AuNRs and RM257 monomer undergo an addition reaction with the sulfhydryl bonds of EDDET.) The reaction is carried out at room temperature and magnetic stirring for 10 h, and then dried in an oven at 80 ℃ to remove the solvent. Finally, the AuNRs@LCE electrostatic spinning solution (the 1st step cross-linking solution system) is generated by dissolving the above product and the photoinitiator (HHMP) in the CH_2Cl_2 solvent.

Fabrication of AuNRs@LCE Yarn Soft Actuator: In this experiment, the AuNRs@LCE yarn soft actuator was manufactured through using molecular synthesis and electrospinning method. The acquired AuNRs@LCE spinning fluids were placed in two syringes. The self-made electrostatic spinning device mainly consists of high voltage power supply (obtained from Tianjin Dongwen High Voltage Power Supply Co.), syringe, metal funnel receiver, PTFE winding roller. During the electrospinning process, two spinnerets were maintained at positive (10 kV) and negative potentials (−8 kV), respectively, and the metal funnel-shaped receiver was kept grounded, a uniform flow rate of 1.2 ml h^{-1} using an infusion pump was applied to polymeric solutions taken in a syringe at a concentration of 24 *wt*%, yielding composite yarns with micro/nano-sized AuNRs@LCE fibers. Both the spinneret was maintained at a relative angle of 45° with respect to metal funnel collector axis, and the distance between the spinnerets and the receiver is 6~8 cm. When positive and negative voltages are applied to the two needles separately, an electrostatic induced effect occurs because the metal spinning funnel in the middle is grounded, causing the micro/nanofibers ejected from the two needles to collect at the edge of the metal funnel. Subsequently, by collecting traction through the glass rod, a spinning triangular cone will be formed at the flare of the metal funnel. By stretching and pulling, oriented nanofiber bundles are formed on the spinning triangle cone, and the rotation of the metal circular target twists the nanofiber bundles on the yarn surface, which is eventually towed and wound onto a circular roller to form different LCE composite yarn. At the same time, the LCE fibers is irradiated by

ultraviolet lamp with wavelength of 365 nm (5mW cm^{-2}) during the spinning process, so that the micro-nano fiber junction goes through the second step of cross-linking reaction. After that, the electrospun AuNRs@LCE micro/nanfiber yarn is formed on the surface of the rotating metal horn by using electrospinning technique. After that, the electrospun AuNRs@LCE micro/nanofiber yarn was stretched to different times (0-14) of their initial length through regulating different rotation speeds of the two rollers, and the stretched fibers are collected on the surface a second PTFE rotating cylinder. The stretched LCE fibers are irradiated with an ultraviolet lamp (30 mW cm^{-2}) with a wavelength of 365 nm for 30 min to make the liquid crystal elastomer fiber undergo the 2nd step cross-linking reaction, so as to fix the arrangement direction of liquid crystal units and molecular chains of the LCE fiber. During the UV cross-linking process, the remaining AuNRs monomers undergo an addition reaction with the carbon-carbon double bonds inside the liquid crystal molecular chains, resulting in the formation of mildly cross-linked liquid crystal elastomers. Finally, scalable liquid crystal yarns with good orientation characteristics and uniform diameter are efficiently and stably manufactured.

1.3.Measurements and Characterization

A scanning electron microscope (SEM, S4800) and elemental energy spectrometer (EDX) were applied to determine micro-morphological structure and element distribution of different electrospun AuNRs@LCE yarn. Transmission electron microscopy (TEM), UV-visible spectrophotometer and X-ray photoelectron spectroscopy (XPS, PHI Quantera II) were implemented to characterize the morphological and chemical structural changes during the synthesis process of AuNRs monomers. Fourier transform infrared spectroscopy (FTIR) and differential scanning calorimetry (DSC) were used to measure the chemical structure and phase transition temperature of various electrospun AuNRs@LCE yarn. Thermogravimetric analysis (TGA) was carried out to investigate the thermal stability of different samples. Polarizing microscope (POM) were used to identify the molecular crystallinity and orientation of various liquid crystal elastomer fibers. A uniaxial testing machine (PT-1198 GDT) was used to measure the tensile properties of different electrospun yarn samples. At the same time, the tensile rate was set to 10 mm min^{-1}, the distance between gauges was 50 mm, and five samples for each specification were used for an average. Before testing the tensile properties

of the yarns, we determined the cross-sectional diameters of the different yarn samples using a scanning electron microscope and calculated their fibre cross-sectional areas, respectively. In addition, to investigate the effect of different strain rates on the mechanical properties of yarns, the breaking strength and fracture strain of the AuNRs@LCE yarn were measured under tensile rate conditions of 5, 10, 15, 20, and 30 mm min^{-1}, respectively. The UV light (365 nm) irradiation were carried out with LED lamps (FUV-6BK, Bangwo Elec. Technologies Co., Ltd., China). The NIR light irradiation was performed by an 808-nm laser (MDL-N-808-10W, Changchun Laser Optoelectronics Technology Co., Ltd., China). Photographs and movies were taken with a digital camera (Canon 80D, Japan). The electrospun AuNRs@LCE micro/nanofibers actuator was modeled as a hyperplastic material. Their performance was set according to the previous mechanical test results. The micro/nanofiber was modeled as an elastic isotropic material with an elastic modulus of 2.8×10^{6} MPa and Poisson's ratio of 0.42 [2, 3]. Considering the actual distribution of fibers in LCE based yarns actuator, the contact relationship between micro and nano fibers is set for stable bonding. The model contained 6499 nodes and 1246 elements after automatic meshing.

1.4. Actuation performances of electrospun AuNRs@LCE yarn actuator

Thermal actuate properties: To measure the thermal actuation properties of different electrospun AuNRs@LCE yarn actuator, the yarn actuator was first placed in a constant temperature oven at different temperatures. It was held at each temperature for 5 minutes until the temperature inside the chamber was stable. Then, we took images with a digital camera (Canon 80D) and characterized the length and actuating deformation rate of the LCE microfiber under different temperature by using the Kinovea software to analyze the images. The actuation strain is calculated as illustrated in **Formula S1** [4].

$$S = \frac{L_0 - L_1}{L_0} \times 100\% \qquad (1)$$

Where $\boldsymbol{S}$ present actuation strain, $\boldsymbol{L_0}$, $\boldsymbol{L_1}$ represents the initial length before heating and the final length after heating of LCE microfibre, respectively.

Light actuate properties: To investigate NIR photothermal actuating properties of electrospun AuNRs@LCE yarn actuator, the photothermal actuation strain and temperature

changes of different electrospun AuNRs@LCE yarn were recorded by a digital camera (Canon 80D) and an infra imaging system (IRC, R500Ex-Pro-D, Japan) under 808 nm NIR light irradiation.

1.5. Fabrication of the light-controlled artificial muscle, micro swimmer and smart hemostatic bandage

Firstly, we created the artificial arm model and fixed the designed AuNRs@LCE yarn soft actuator to simulate the artificial muscle elements at both ends of the model. In this work, the contraction and recovery of light controlled artificial muscle fibers were realized by regulating the near-infrared light of different energies. Meantime, a Canon camera was used to record the movement process of artificial muscle driven by NIR light of different powers, and the change of its contraction angle versus time was analyzed. Secondly, the light-driven micro swimmer was composed of a bending structure made from polyethylene terephthalate (PET) film, polyimide tape (with curved ends), and AuNRs@LCE yarn. In the experiment, two polyimide tapes were glued at the front and rear sides of miniature swimmer model. At the same time, the obtained AuNRs@LCE yarn soft actuator was mounted in the middle to trigger the foldable structure. Finally, we prepared a novel intelligent hemostatic bandage based on the as-prepared AuNRs@LCE yarn soft actuator by plain weaving strategy. Moreover, a simple model was designed and constructed to simulate the working process of the optically controlled hemostatic bandage, which mainly consisted of an injection pump, an arm model, a plastic hose, an 18-gauge industrial needle and a precision electronic balance. In the study, a plastic hose was attached to the surface of the arm model, and the generated hemostatic bandage was then wound and fixed to the arm model. When NIR light irradiates a hemostatic bandage, the liquid crystal fibers inside the bandage gradually contract and deform due to the photothermal effect, thereby slowing down the flow rate of the red solution. Finally, the hemostatic effect of the bandage is calculated by recording real-time weight values on a precision electronic balance.

2. Supplementary Figures and Table

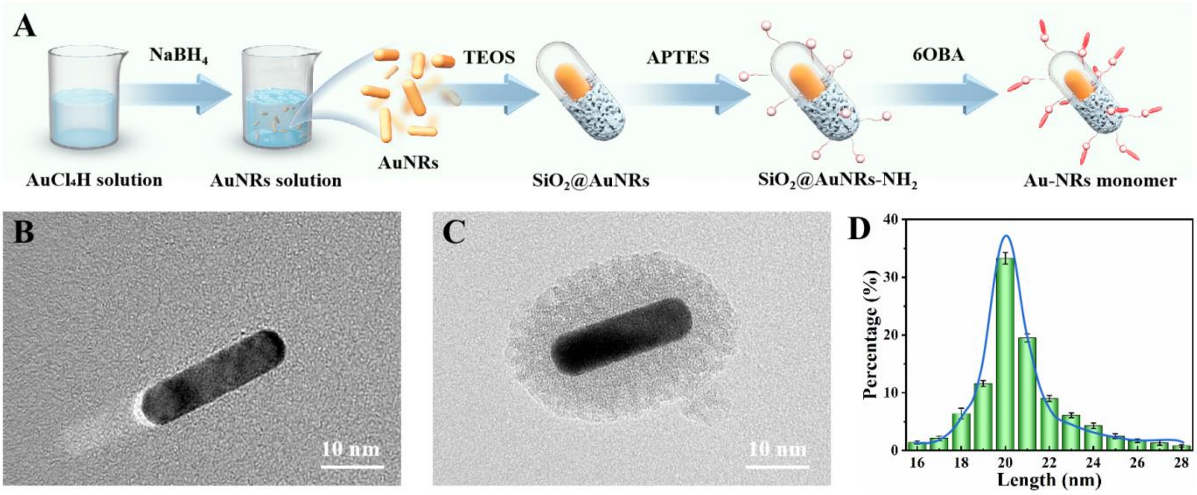

Figure S1. Synthesis schematic diagram and microstructure of polymerizable functional AuNRs monomer. A) The preparation diagrammatic sketch of polymerizable AuNRs nanomonomer. B-C) TEM images of AuNRs monomers before and after modification, respectively. D) The length distribution of AuNRs particles.

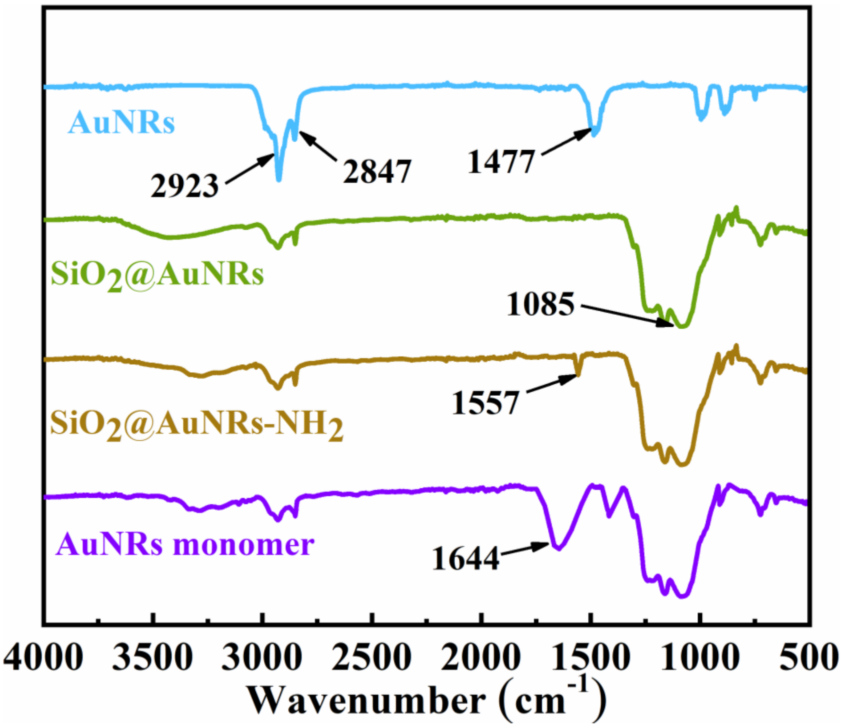

Figure S2. Fourier transform infrared spectra of different AuNRs samples.

Figure S2 depicted that the absorption peaks at 2923 cm^{-1} and 2847 cm^{-1} are attributed to the C-H stretching vibration of hexadecyl trimethyl ammonium bromide (CTAB) on the surface of AuNRs. The absorption peak at 1477 cm^{-1} is attributed to the asymmetric C-H vibration of CTAB (CH^3-N^+). Furthermore, the Si-O-Si characteristic absorption peak is formed on the surface of SiO_2@AuNRs at 1085 cm^{-1} after modification with SiO_2 coating. The feature absorption peaks at 1557 cm^{-1} (-N-H) implied the successful salinization grafting of (3-aminopropyl)triethoxysilane (APTES) on SiO_2@AuNRs, while the feature absorption peaks at 1644 cm^{-1} (-C=O) indicated formation of the covalent bonding between the SiO_2@AuNRs-NH_2 and 4-((6-(acryloyloxy) hexyloxy) benzoic acid (6OBA). Results clearly indicated that grafting of -NH_2 onto the SiO_2@AuNRs surface and subsequent formation of the covalently-bonded AuNRs nanomonomer.

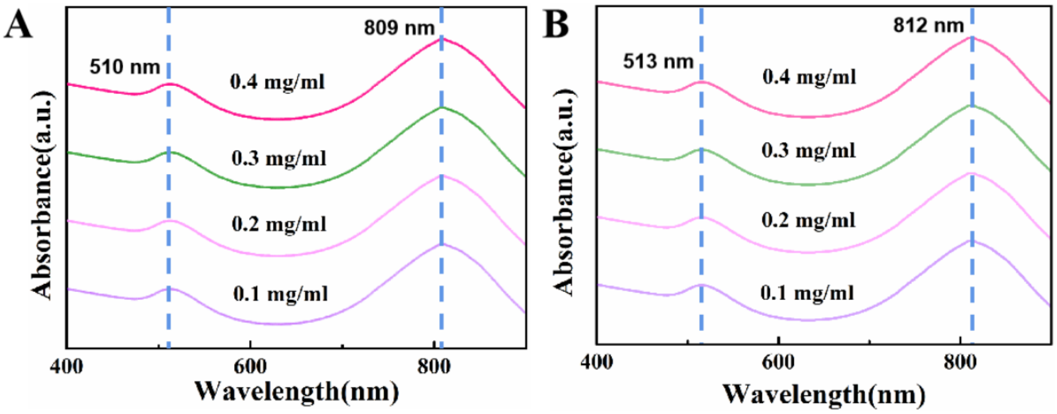

Figure S3. A-B) Ultraviolet-visible absorption spectra of AuNRs and AuNRs monomer in aqueous solution, respectively.

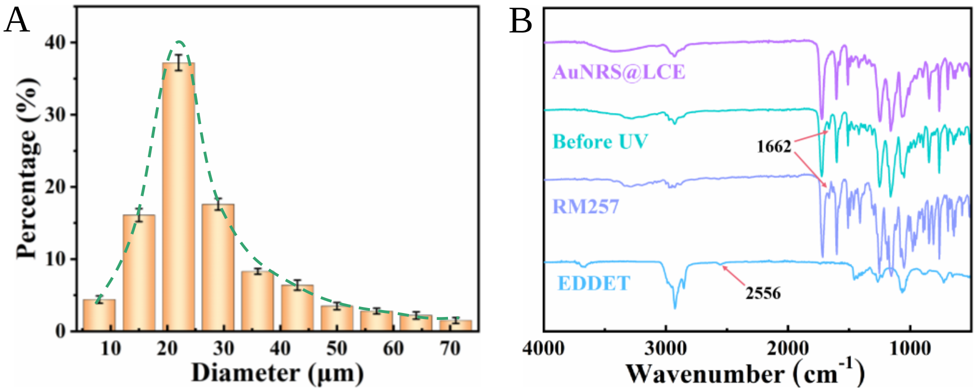

Figure S4. A) Diameter and size distribution diagram of electrospun AuNRs@LCE fibers. B) Fourier transform infrared spectra of different samples.

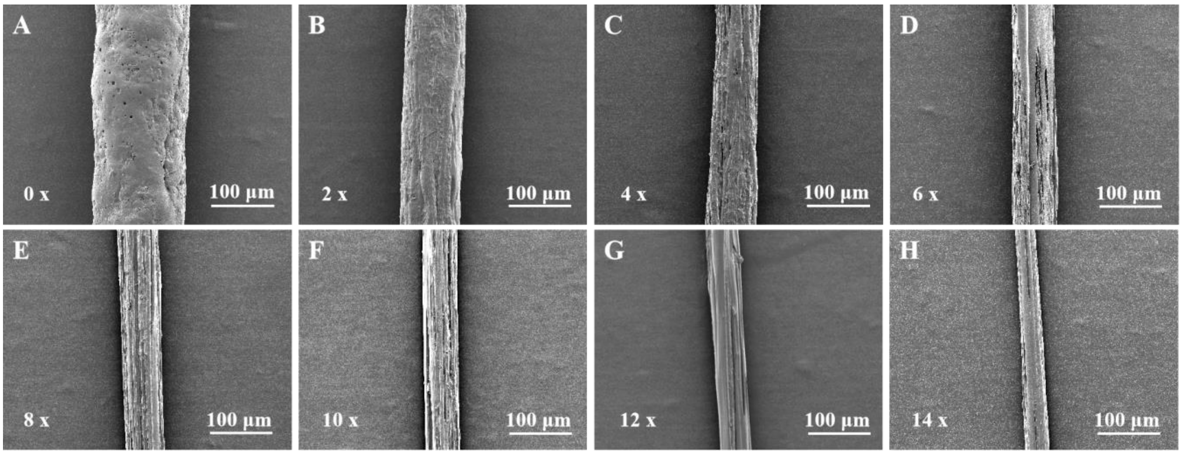

Figure S5. A-H) The surface morphology of the designed yarn soft actuator after stretch at different multiples (0-14) and two-step crosslinking treatment strategy.

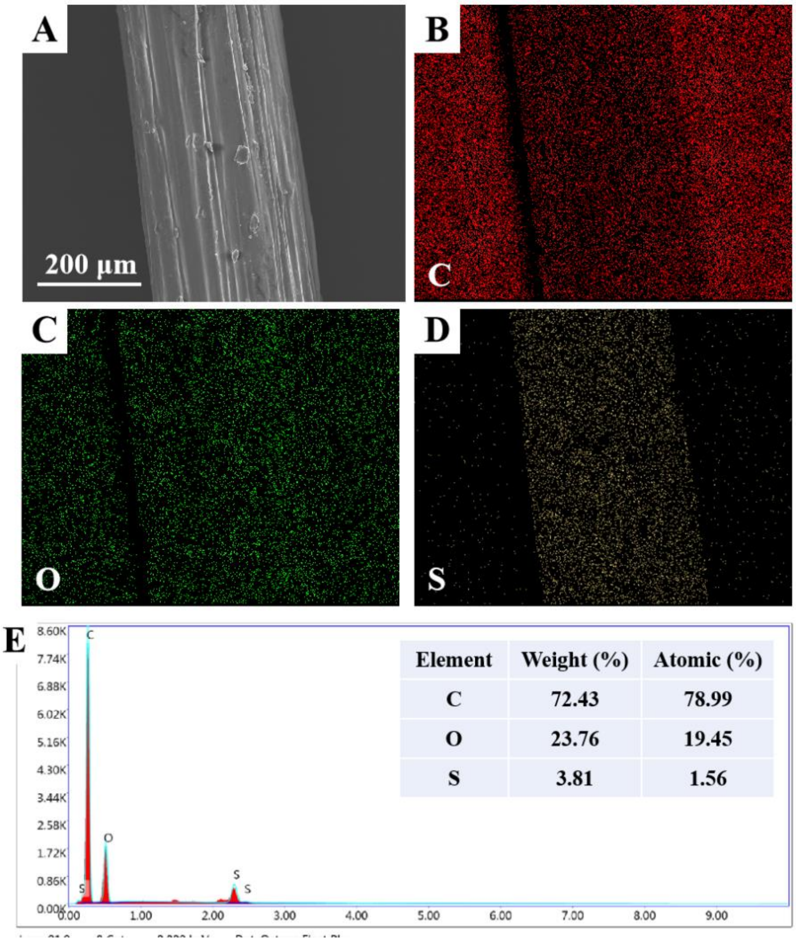

Figure S6. A-E) The microscopic morphology and elemental energy spectrum distribution diagram of electrospun LCE active yarn.

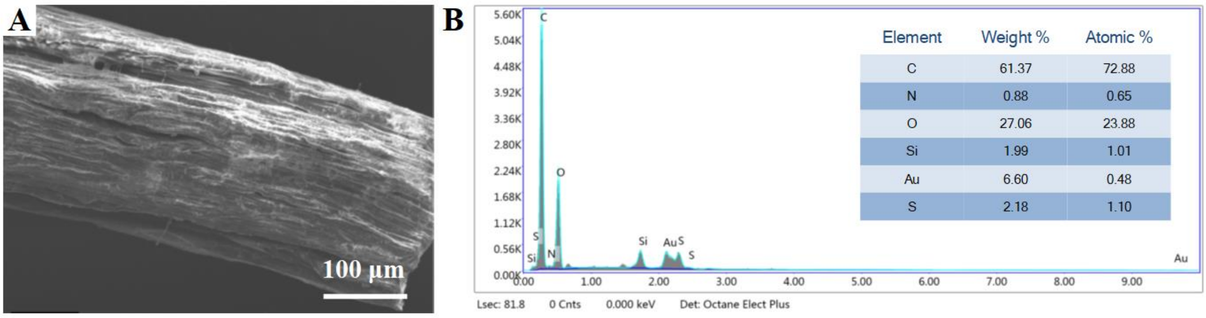

Figure S7. A-B) The microscopic morphology and elemental energy spectrum distribution diagram of electrospun AuNRs@LCE active yarn.

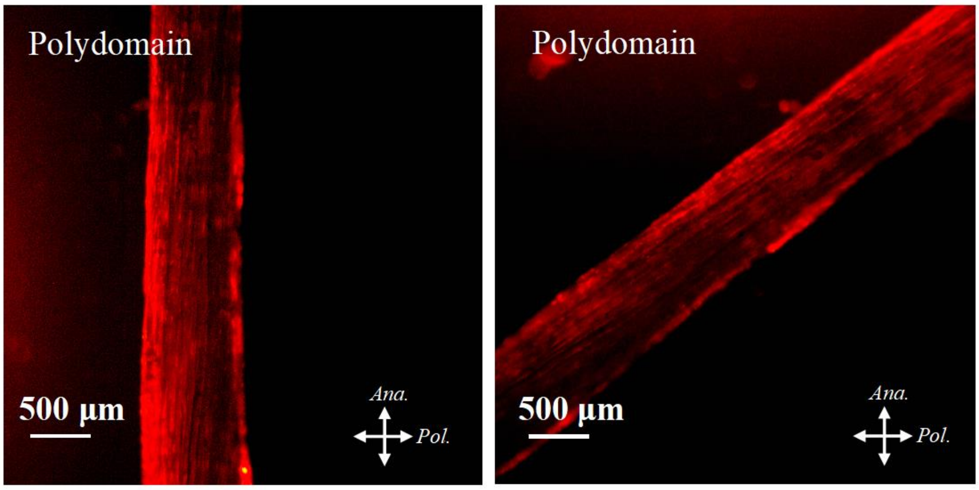

Figure S8. The POM image of the electrospun polydomain AuNRs@LCE active yarn viewed at two different angles with respect to the analyzer.

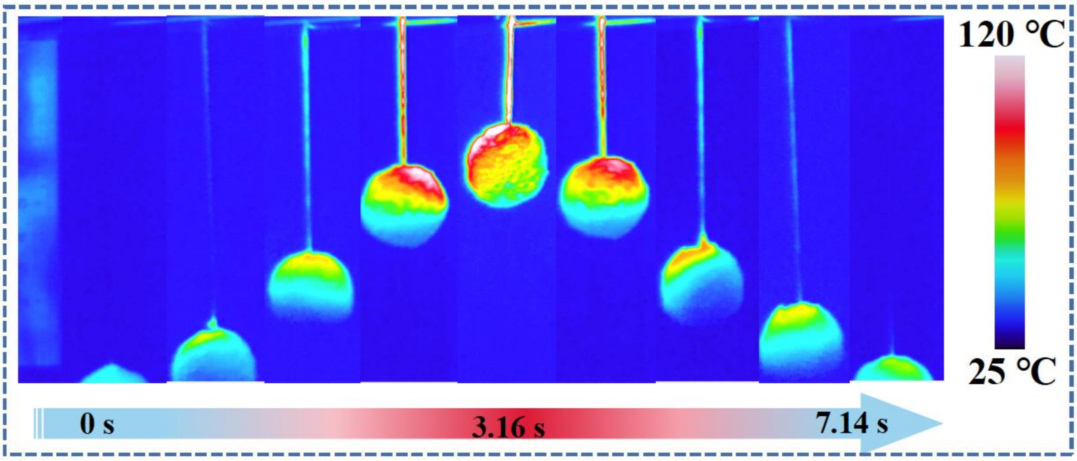

Figure S9. Infrared images of AuNRs@LCE yarn actuator lifting a round ball (400 mg)

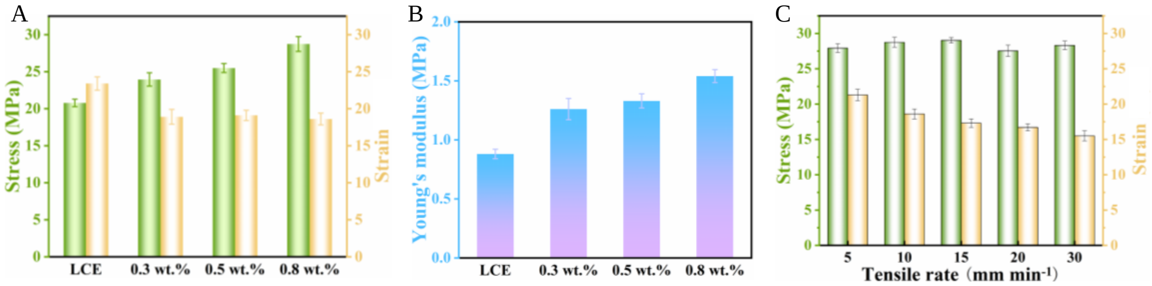

Figure S10. A-B) Breaking strength, strain and Young's modulus of different AuNRs@LCE active yarn. C) Mechanical properties of AuNRs@LCE active yarn (0.8 wt.%) yarns under different tensile rate.

.

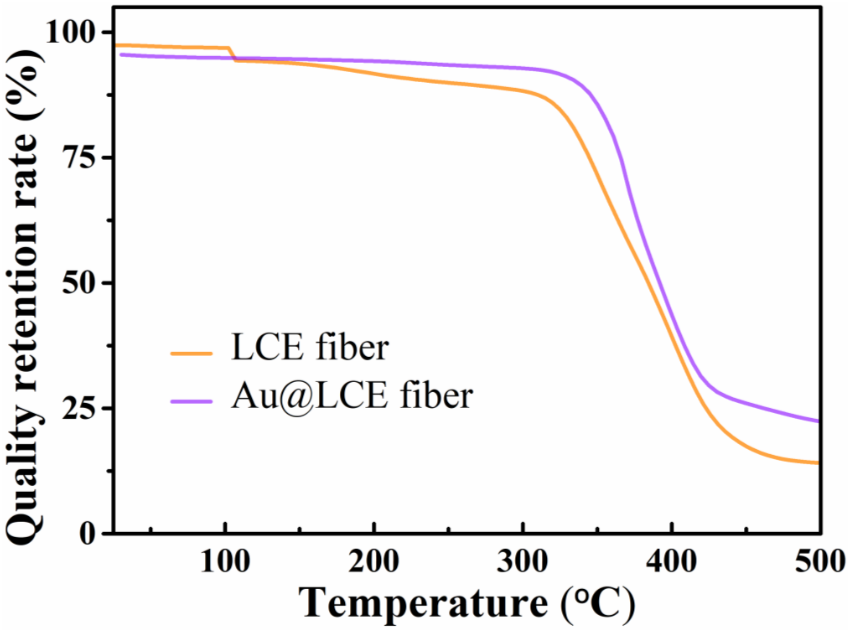

Figure S11. Thermal stability performances of LCE and Au-NRs@LCE active yarn.

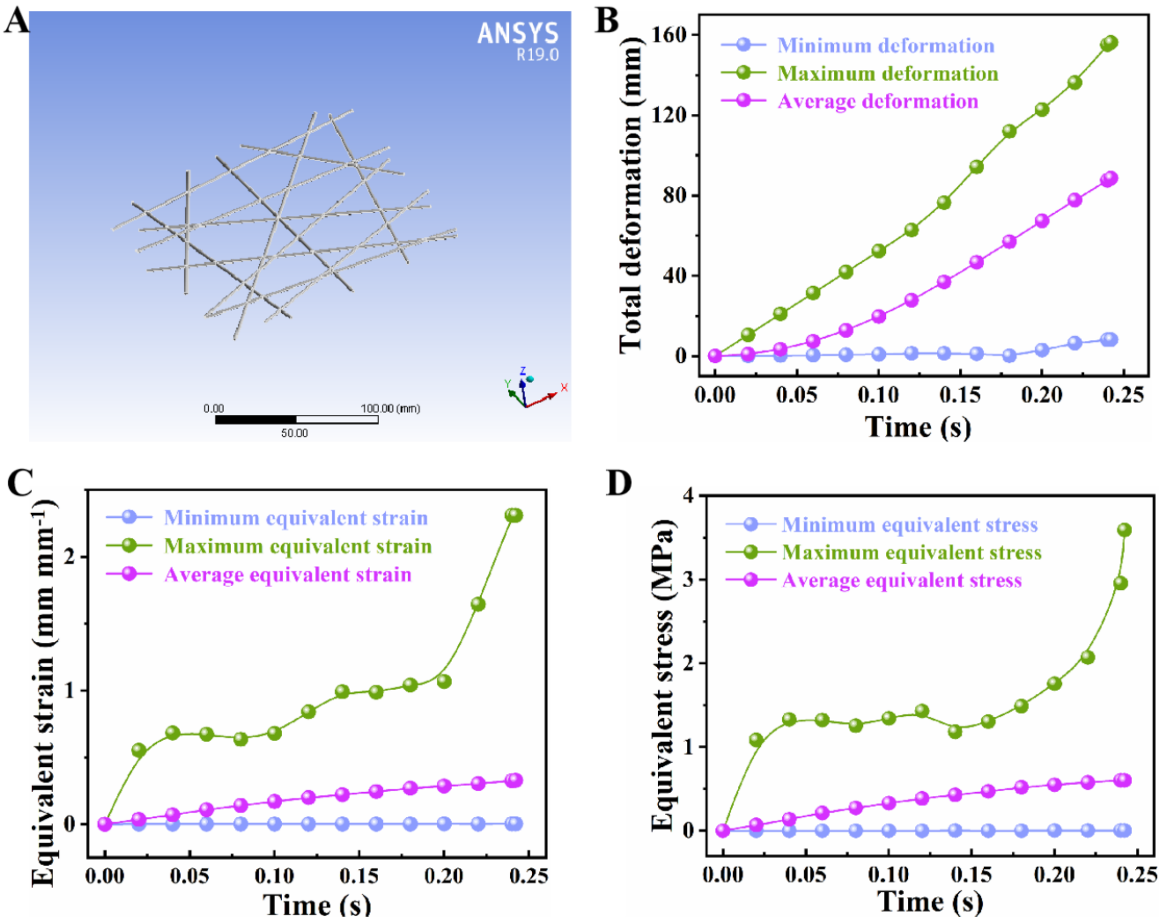

Figure S12. A) The schematic diagram of AuNRs@LCE fiber network model. B-D) The variation curves of total strain, equivalent strain, and equivalent stress over time, respectively.

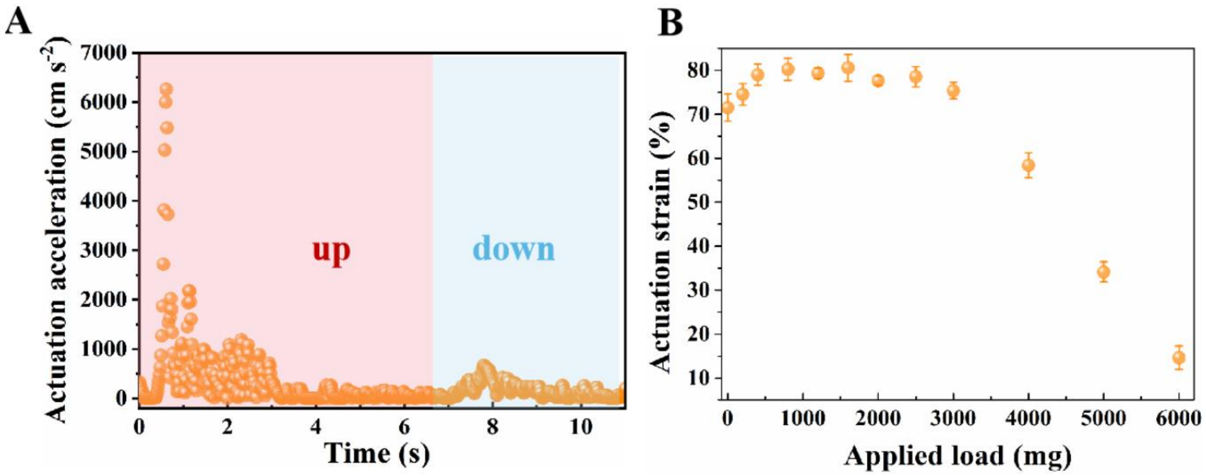

Figure S13. A-B) The thermal actuation acceleration and lifting weight of AuNRs@LCE yarn soft actuator.

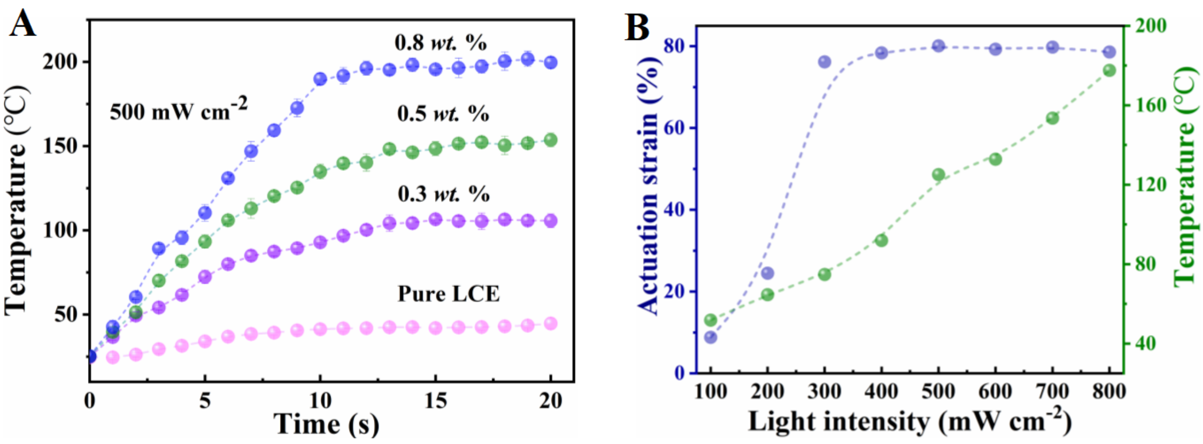

Figure S14. A) The relationship between photo-thermal temperature and time for yarn actuator with different AuNRs content. B) The photo-thermal temperature and actuation strain of the yarn actuator under different light intensities.

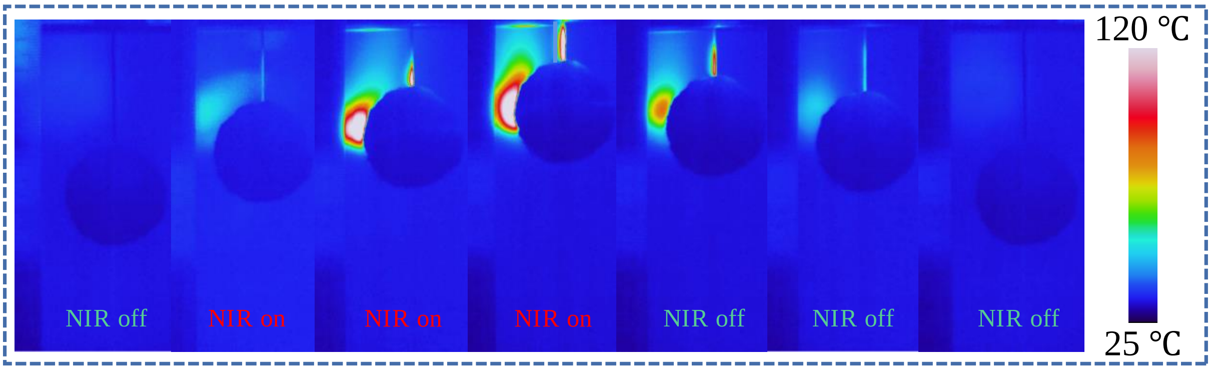

Figure S15. Infrared images of NIR light controlled AuNRs@LCE yarn actuator lifting a round ball (600 mg).

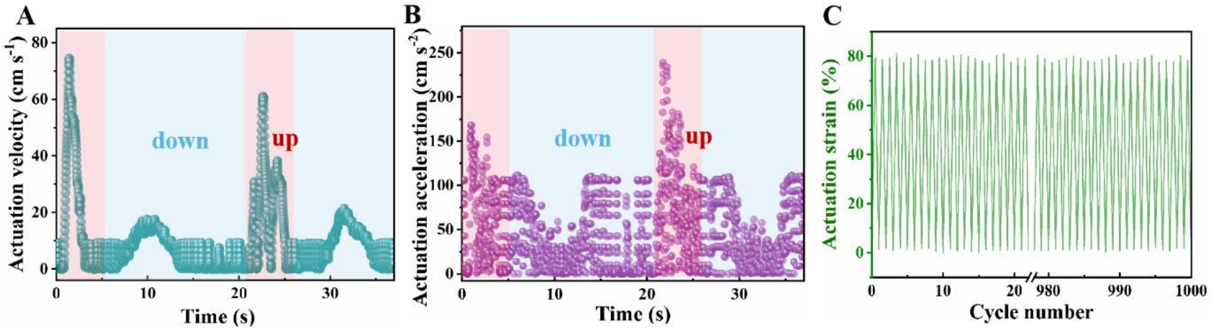

Figure S16. A-C) The NIR light actuation speed, acceleration and reusability of AuNRs@LCE active yarn actuator.

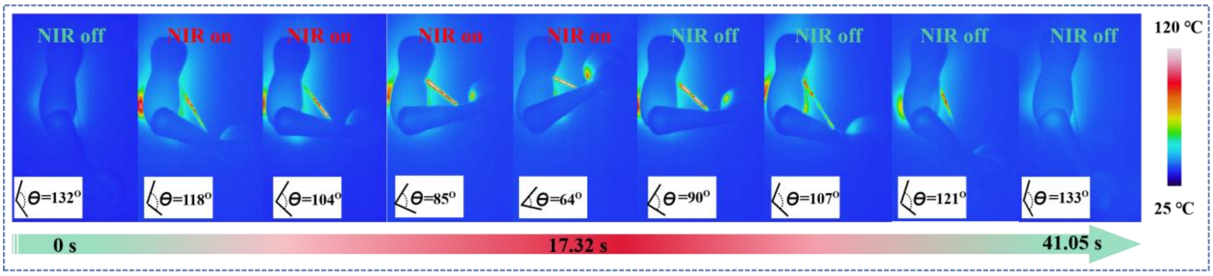

Figure S17. Infrared images of artificial muscle model based on AuNRs@LCE yarn actuator lifting a dumbbell model (3000 mg).

.

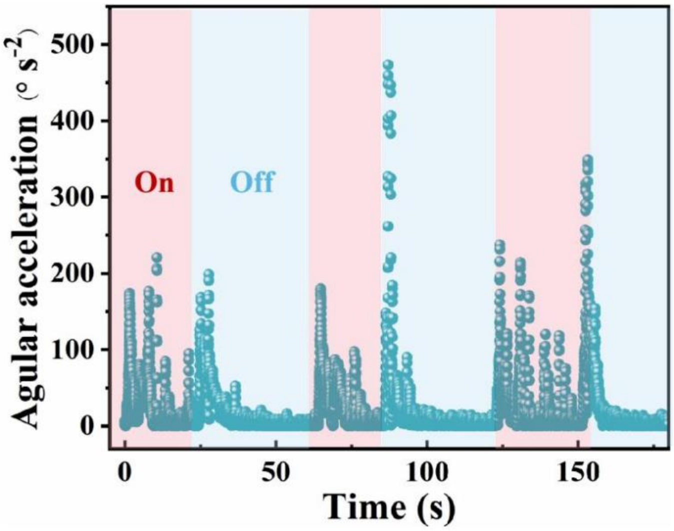

Figure S18. The NIR light driving angle acceleration of artificial muscle model based on the AuNRs@LCE yarn soft actuator.

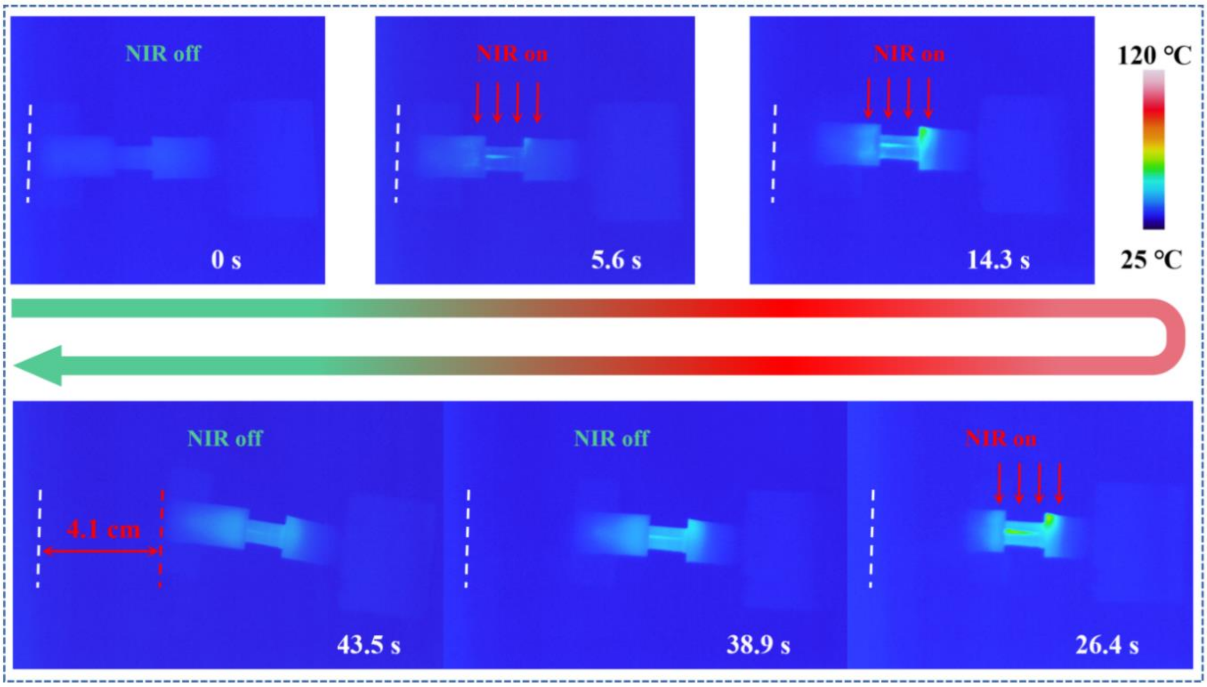

Figure S19. Infrared images of the swimming process of a NIR light controlled micro swimmer based on the AuNRs@LCE yarn soft actuator.

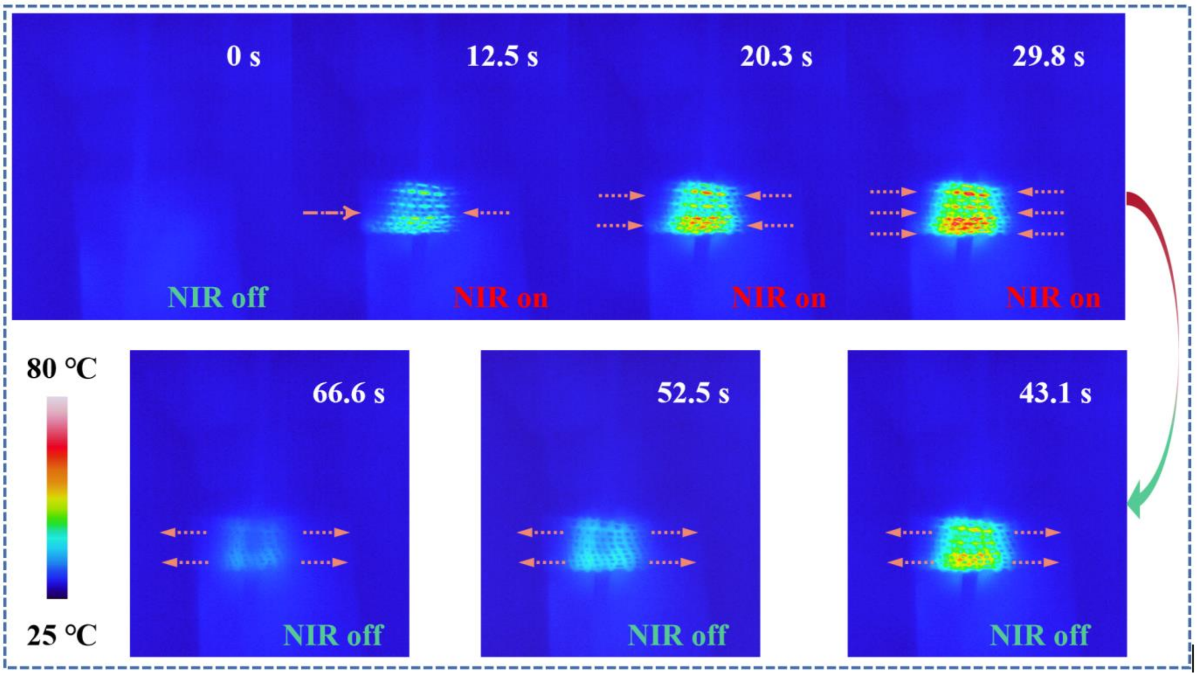

Figure S20. Infrared images of light-controlled smart bandage hemostasis process based on the Au-NRs@LCE yarn soft actuator.

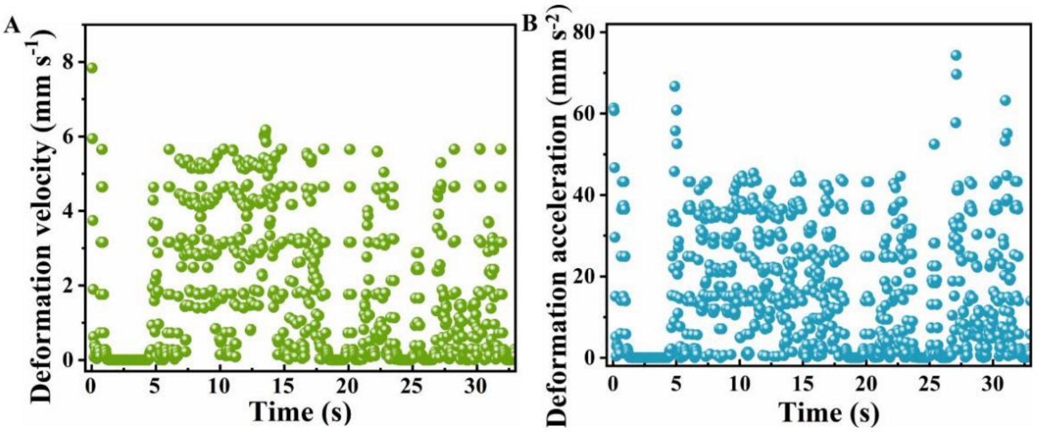

Figure S21. A-B) The deformation speed and acceleration of the infusion tube under the contraction of the designed hemostatic bandage.

Table S1. Actuation performances and applications of the electrospun AuNRs@LCE yarn soft actuator in comparison with reported literature.

Materials	Method & strategy	Actuate strain	Actuate time	Reuse ability	Application
LM-LCE composite fiber [5]	dry spinning	40 %	10 s	1000	electronically control artificial muscle
LM-LCE fiber [6]	4D printable	5-20 %	15-180 s	N/R	NIR light control switch
LCE fibers [7]	direct ink write printing	51 %	20-60 s	N/R	thermal control artificial muscle
LM-LCE coaxial fiber [8]	3D direct ink printing	50 %	20 s	25	self-sensing 3D soft robot
PDA-coated LCE microfiber [9]	electrospun technique	10-55 %	0.2-0.4 s	1000	microtweezer, microswimmer, microsoft pump
LLCP fibers [10]	shaping drafting technique	80 %	16 s	N/R	smart hinges, spider webs
CNTs/LCE core-shell fibers [11]	shaping drafting technique	20-45 %	8-50 s	50	artificial muscle， optical gripper
PDA@MXene/LCE fiber [12]	dry spinning	55 %	0.4-5 s	1000	intelligent window, photothermal circuit switch
Twisted LCE fiber [13]	shaping drafting technique	40 %	4 s	100	rotating microengines
LCE actuator [14]	melt spinning	34 %	4-6 s	50	knitting intelligent fabric actuator
LCE yarn [15]	dry spinning	30-41%	5-20 s	5	smart textiles
LCE/graphene [16]	draft spinning and photocrosslinking	15 %	0.25 s	25000	light controlled artificial muscle
Gold coated LCE [17]	compression assisted molding and magnetron sputter	40-50 %	0.2-0.4 s	1000	motor unit, electrically controlled artificial muscle
LCE/ PU network [18]	molecular synthesis and stretching	17-20 %	600-900 s	N/R	hand-shape actuator

CNTs@LCE [19]	stretch molding	45 %	25-50 s	100	artificial flower, serpentine actuator
**AuNRs@LcE yarn actuator (This work)**	**electrospun technique**	**81 %**	**3.1-4.9 s**	**1000**	**light-controlled artificial muscle, micro swimmer, hemostatic bandage**

3. Captions of Supplementary Movies

Supplementary Video S1: Schematic of the Preparation Process of Electrospun AuNRs@LCE Active Yarn.

Supplementary Video S2: Dynamic Images of AuNRs@LCE Active Yarn Polarizing Microscope.

Supplementary Video S3: Thermal Actuation Process of the AuNRs@LCE Yarn Soft Actuator.

Supplementary Video S4: Finite Element Simulation of the Total Strain-Time of Electrospun AuNRs@LCE Fibers Network During Stretching.

Supplementary Video S5: The AuNRs@LCE Yarn Soft Actuator Driven by 808 nm NIR Light.

Supplementary Video S6: NIR Light-Controlled Artificial Muscle Constructed with the AuNRs@LCE Yarn Soft Actuator.

Supplementary Video S7: NIR Light-Controlled Micro Swimmer Constructed with the AuNRs@LCE Yarn Soft Actuator.

Supplementary Video S8: NIR Light-Controlled Smart Hemostatic Bandage Constructed with the AuNRs@LCE Yarn Soft Actuator.

References

[1] X. Yang, Y. Chen, X. Zhang, P. Xue, P. Lv, Y. Yang, L. Wang, W. Feng, *Nano Today* **2022**, *43*, 101419.

[2] Y. Li, C. Luo, K. Yu, X. Wang, *ACS Appl. Mater. Interfaces* **2021**, *13*, 8929-8939.

[3] J. Jia, Y. Peng, X. J. Zha, K. Ke, R. Y. Bao, Z. Y. Liu, M. B. Yang, W. Yang, *ACS Nano* **2022**, *16*, 16806-16815.

[4] C. Lang, E. C. Lloyd, K. E. Matuszewski, Y. Xu, V. Ganesan, R. Huang, M. Kumar, R. J. Hickey, *Nat. Nanotechnol.* **2022**, *17*, 752-758.

[5] J. Sun, Y. Wang, W. Liao, Z. Yang, *Small* **2021**, *17*, e2103700.

[6] C. P. Ambulo, M. J. Ford, K. Searles, C. Majidi, T. H. Ware, *ACS Appl. Mater. Interfaces* **2021**, *13*, 12805-12813.

[7] D. J. Roach, C. Yuan, X. Kuang, V. C. Li, P. Blake, M. L. Romero, I. Hammel, K. Yu, H. J. Qi, *ACS Appl. Mater. Interfaces* **2019**, *11*, 19514-19521.

[8] A. Kotikian, J. M. Morales, A. Lu, J. Mueller, Z. S. Davidson, J. W. Boley, J. A. Lewis, *Adv. Mater.* **2021**, *33*, e2101814.

[9] Q. He, Z. Wang, Y. Wang, Z. Wang, C. Li, R. Annapooranan, J. Zeng, R. Chen, S. Cai, *Sci. Robot*. **2021**, *6*, eabi9704.

[10] X. Pang, L. Qin, B. Xu, Q. Liu, Y. Yu, *Adv. Funct. Mater.* **2020**, *30*, 2002451.

[11] Y. Yu, L. Li, E. Liu, X. Han, J. Wang, Y.-X. Xie, C. Lu, *Carbon* **2022**, *187*, 97-107.

[12] D. S. Wu, Y. N. Zhang, H. R. Yang, A. F. Wei, Y. X. Zhang, A. Mensah, R. Yin, P. F. Lv, Q. Feng, Q. F. Wei, *Mater. Horiz.* **2023**, *10*, 2587-2598.

[13] Y. Wang, J. Sun, W. Liao, Z. Yang, *Adv. Mater.* **2022**, *34*, e2107840.

[14] J. Sun, W. Liao, Z. Yang, *Adv. Mater.* **2023**, *35*, e2302706.

[15] P. E. S. Silva, X. Lin, M. Vaara, M. Mohan, J. Vapaavuori, E. M. Terentjev, *Adv. Mater.* **2023**, *35*, e2210689.

[16] W. Hou, J. Wang, J. A. Lv, *Adv. Mater.* **2023**, *35*, e2211800.

[17] Y. Wang, Q. He, Z. Wang, S. Zhang, C. Li, Z. Wang, Y. L. Park, S. Cai, *Adv. Mater.* **2023**, *35*, e2211283.

[18] C. Song, Y. Zhang, J. Bao, Z. Wang, L. Zhang, J. Sun, R. Lan, Z. Yu, S. Zhu, H. Yang, *Adv. Funct. Mater*. **2023**, *33*, 2213771.

[19] J. Zhang, D. Sun, B. Zhang, Q. Sun, Y. Zhang, S. Liu, Y. Wang, C. Liu, J. Chen, J. Chen, Y. Song, X. Liu, *Mater. Horiz.* **2022**, *9*, 1045-1056.
